# Ancient Genomics Reveals the Origin, Dispersal, and Human Management of East Asian Domestic Pigs

**DOI:** 10.1093/molbev/msaf214

**Published:** 2025-09-25

**Authors:** Yu Han, Zhihan Zhao, David W G Stanton, Xiao-Le Lei, Zhuang Wu, Yiting Liu, Chong Yu, Xi Chen, Wenyan Li, Juan Wang, Yue You, Yue Li, Sha Lei, Hailin Yi, Wenquan Fan, Quanfa Cai, Rui Min, Changcheng Hu, Canping Chen, Yingjie Cui, Jiqiao Guo, Hongliang Zhang, Haichao Song, Xin Guo, Qiurong Ruan, Yuhua Tan, Ziyi Li, Xiangyu Zhang, Xingyu Shi, Xu Zhou, Yan Zhuang, Aurélie Manin, Laurent A F Frantz, Joel M Alves, Yan Pan, Xiaohong Wu, Shu-Jin Luo, Greger Larson, He Yu

**Affiliations:** State Key Laboratory of Gene Function and Modulation Research, School of Life Sciences; Institute of Ecology, Peking University, Beijing 100871, China; The Palaeogenomics & Bio-Archaeology Research Network, Research Laboratory for Archaeology and History of Art, University of Oxford, Oxford OX1 3QY, UK; State Key Laboratory of Gene Function and Modulation Research, School of Life Sciences; Institute of Ecology, Peking University, Beijing 100871, China; Peking University-Tsinghua University-National Institute of Biological Sciences Joint Graduate Program, Academy for Advanced Interdisciplinary Studies, Peking University, Beijing 100871, China; School of Biosciences, Cardiff University, Cardiff CF10 3AX, UK; State Key Laboratory of Gene Function and Modulation Research, School of Life Sciences; Institute of Ecology, Peking University, Beijing 100871, China; School of Life Sciences, Tsinghua University, Beijing 100084, China; Peking University-Tsinghua University-National Institute of Biological Sciences Joint Graduate Program, School of Life Sciences, Tsinghua University, Beijing 100084, China; School of History and Culture, Hebei Normal University, Shijiazhuang 050024, China; School of History, Wuhan University, Wuhan 430072, China; School of Sociology and Anthropology, Sun Yat-sen University, Guangzhou 510275, China; Department of Cultural Heritage and Museology, Nanjing Normal University, Nanjing 210023, China; Hebei Culture Relics Bureau, Shijiazhuang 050021, China; Department for the History of Science and Scientific Archaeology, University of Science and Technology of China, Hefei 230026, China; School of History, Capital Normal University, Beijing 100089, China; School of Cultural Heritage, Northwest University, Xi'an 710069, China; Department of Cultural Heritage and Museology, Fudan University, Shanghai 200433, China; Department of Archaeology, Durham University, Durham DH1 3LE, UK; Henan Provincial Institute of Cultural Heritage and Archaeology, Zhengzhou 450099, China; Henan Provincial Institute of Cultural Heritage and Archaeology, Zhengzhou 450099, China; Yunnan Province Institute for Cultural Relics and Archaeology, Kunming 650118, China; Yunnan Province Institute for Cultural Relics and Archaeology, Kunming 650118, China; School of History, Culture and Tourism, Guangxi Normal University, Guilin 541001, China; School of History and Culture, Hebei Normal University, Shijiazhuang 050024, China; School of History and Culture, Hebei Normal University, Shijiazhuang 050024, China; Yulin Archaeological Exploration Team of Shaanxi Province, Yulin 719000, China; Hebei Provincial Institute of Cultural Relics and Archaeology, Shijiazhuang 050031, China; School of History and Culture, Luoyang Normal University, Luoyang 471000, China; Luoyang City Cultural Relics and Archaeology Research Institute, Luoyang 471000, China; School of History, Wuhan University, Wuhan 430072, China; Xi'an Institute of Archaeology and Conservation on Culture Heritage, Xi'an 710069, China; Xinjiang Cultural Relics and Archaeology Institute, Ürümchi 830011, China; Sun Yat-sen University Museum, Guangzhou 510275, China; Archaeology and Conservation Scientific Research Institute, National Centre for Archaeology, Bejing 100020, China; Xi'an Institute of Archaeology and Conservation on Culture Heritage, Xi'an 710069, China; State Key Laboratory of Gene Function and Modulation Research, School of Life Sciences; Institute of Ecology, Peking University, Beijing 100871, China; State Key Laboratory of Gene Function and Modulation Research, School of Life Sciences; Institute of Ecology, Peking University, Beijing 100871, China; State Key Laboratory of Gene Function and Modulation Research, School of Life Sciences; Institute of Ecology, Peking University, Beijing 100871, China; Peking-Tsinghua Centre for Life Sciences, Academy for Advanced Interdisciplinary Studies, Peking University, Beijing 100871, China; The Palaeogenomics & Bio-Archaeology Research Network, Research Laboratory for Archaeology and History of Art, University of Oxford, Oxford OX1 3QY, UK; UMR 8096 Archéologie des Amérique, CNRS/Université Paris 1 Panthéon-Sorbonne, Paris 75004, France; Palaeogenomics Group, Institute of Palaeoanatomy, Domestication Research and the History of Veterinary Medicine, Ludwig-Maximilians-Universität München, Munich, Germany; School of Biological and Behavioural Sciences, Queen Mary University of London, London, UK; The Palaeogenomics & Bio-Archaeology Research Network, Research Laboratory for Archaeology and History of Art, University of Oxford, Oxford OX1 3QY, UK; CIBIO, Centro de Investigação em Biodiversidade e Recursos Genéticos, InBIO Laboratório Associado, Universidade do Porto, Vairão 4485-661, Portugal; BIOPOLIS Program in Genomics, Biodiversity and Land Planning, CIBIO, Vairão 4485-661, Portugal; The Key Laboratory for Archaeological Science (The State Ministry of Education), School of Archaeology and Museology, Peking University, Beijing 100871, China; The Key Laboratory for Archaeological Science (The State Ministry of Education), School of Archaeology and Museology, Peking University, Beijing 100871, China; State Key Laboratory of Gene Function and Modulation Research, School of Life Sciences; Institute of Ecology, Peking University, Beijing 100871, China; Peking-Tsinghua Centre for Life Sciences, Academy for Advanced Interdisciplinary Studies, Peking University, Beijing 100871, China; The Palaeogenomics & Bio-Archaeology Research Network, Research Laboratory for Archaeology and History of Art, University of Oxford, Oxford OX1 3QY, UK; State Key Laboratory of Gene Function and Modulation Research, School of Life Sciences; Institute of Ecology, Peking University, Beijing 100871, China

## Abstract

Pigs are the most commercially important modern livestock animal in East Asia. Numerous aspects of their domestication history remain unclear, however, including the geographic center of their domestication, their subsequent dispersal routes, and the emergence of phenotypic traits specific to domestic pigs. To address these questions, we generated 21 nuclear genomes and 23 mitogenomes from ancient domestic pigs and wild boar from 5,800 BCE to 1,300 CE across China. Our analyses of newly generated and previously published Eurasian suid genomes confirmed Northern China and eliminated Southwestern China as the domestication origin of modern East Asian pigs. Following their association with people and the first appearance of black coat coloration, Northern Chinese domestic pigs dispersed alongside Yellow River millet farmers to the Yangtze River Basin and Southwestern China, which they admixed with local wild boar. A genome-wide loss of diversity and signatures of inbreeding in ancient Northern pigs may have been the result of intensified human management as early as 3,000 BCE. Our results reveal the geographic and temporal origins and subsequent dispersal and admixture of pigs in China, mirroring human migration and agricultural development history.

## Introduction

Pigs (*Sus scrofa*) were independently domesticated from 2 geographically and genetically differentiated wild populations in Western and Eastern Eurasia (Giuffra et al. 2000; Larson et al. 2010; Groenen et al. 2012). In Western Eurasia, archaeological and genetic evidence has shown that the earliest domestic pigs were derived from Near Eastern wild boar in Anatolia ∼10,000 years ago (Ottoni et al. 2013; Frantz et al. 2019). These pigs, derived from Near Eastern wild boar accompanied the expansion of Anatolian farmers into Europe ∼8,000 years ago, after which gene flow with European wild boar led to a near complete genetic turnover of pig ancestry within ∼3,000 years (Frantz et al. 2019). The spatiotemporal origins of pigs in Eastern Eurasia and their subsequent dispersal, however, remain uncertain.

Multiple regions in Eastern Eurasia have been proposed as independent centers of pig domestication. Among them, the most clearly archaeologically supported center is in Northern China, as evidenced by the presence of domestic pigs at the sites of Jiahu (∼6,600 BCE) (Cucchi et al. 2011) and Cishan (∼6,000 BCE) (Yuan and Flad 2002).

Within this region, 2 major shifts in the human-pig relationship, possibly indicating intensified pig management, are evident beginning ∼4,000 BCE. First, the zooarcheological record reveals a significant increase in the proportion of pigs among mammalian remains. Specifically, pigs make up nearly 80% of the minimum number of individuals (MNI), thus highlighting their central role in subsistence strategies (Liu et al. 2024). Second, the evidence from dietary isotopes revealed that pigs moved away from a more variable diet to one dominated by C_4_ plants, including millet (Dong and Yuan 2020; Zhang et al. 2021; You et al. 2024). The intensified millet-pig agriculture system from at least 3,500 BCE may have underpinned the rise of early complex societies in Northern China (Yang et al. 2022).

Domestic pigs dated to ∼6,000 BCE are also present in the Lower Yangtze River Basin in Southeastern China, with evidence from the Kuahuqiao and Jingtoushan sites. This region is considered to have hosted an independent process of pig domestication, given the archaeological contexts and dental calculus residues that contained starch granules and parasite remains similar to domestic pigs (Yuan et al. 2008; Wang et al. 2025).

Southwestern and Southern China have also been suggested as independent centers of pig domestication based on mitochondrial and nuclear genetic ancestries found in modern pigs, though these claims have not been supported by archaeological remains (Wu et al. 2007; Peng et al. 2022). Ancient human genomic studies have demonstrated that the early farmers in Southwestern China from at least 2,000 BCE were descendants of Northern Chinese millet farmers (Yang et al. 2020; C. Wang et al. 2021a; Tao et al. 2023). This suggests that, analogous to the pattern observed in Western Eurasia, the domestic pigs found in Southwestern China may have been introduced from the North, after which they admixed with local wild boar (Wen et al. 2022; Zhang et al. 2022).

Animal domestication is often accompanied by the accumulation of coat color variations that are rare in wild populations (Linderholm and Larson 2013). Native domestic pigs across Eurasia display black (and other non-camouflage) coats associated with different nonsynonymous mutations in the Melanocortin 1 Receptor gene (*MC1R*) gene (Linderholm et al. 2016). In Western Eurasia, one of the earliest pieces of evidence of black coat color was found in an Anatolian domestic pig at the site of Ulucak Höyük (∼6,700 BCE) (Frantz et al. 2019). Modern pigs in Eastern Eurasia and Polynesia possess 2 different *MC1R* mutations that lead to black coat color (Fang et al. 2009; Linderholm et al. 2016), though when and where these haplotypes first appeared is unknown.

Here, we began by addressing the geographical origins of domestic pigs in East Asia and their subsequent dispersal across China. We then tested whether the intensified pig management in Northern China, as suggested by the zooarchaeological and isotopic evidence, was corroborated by ancient pig genomic signatures. Lastly, we assessed the temporal pattern of black coat color variability in wild boar and domestic pigs.

**Fig. 1.**
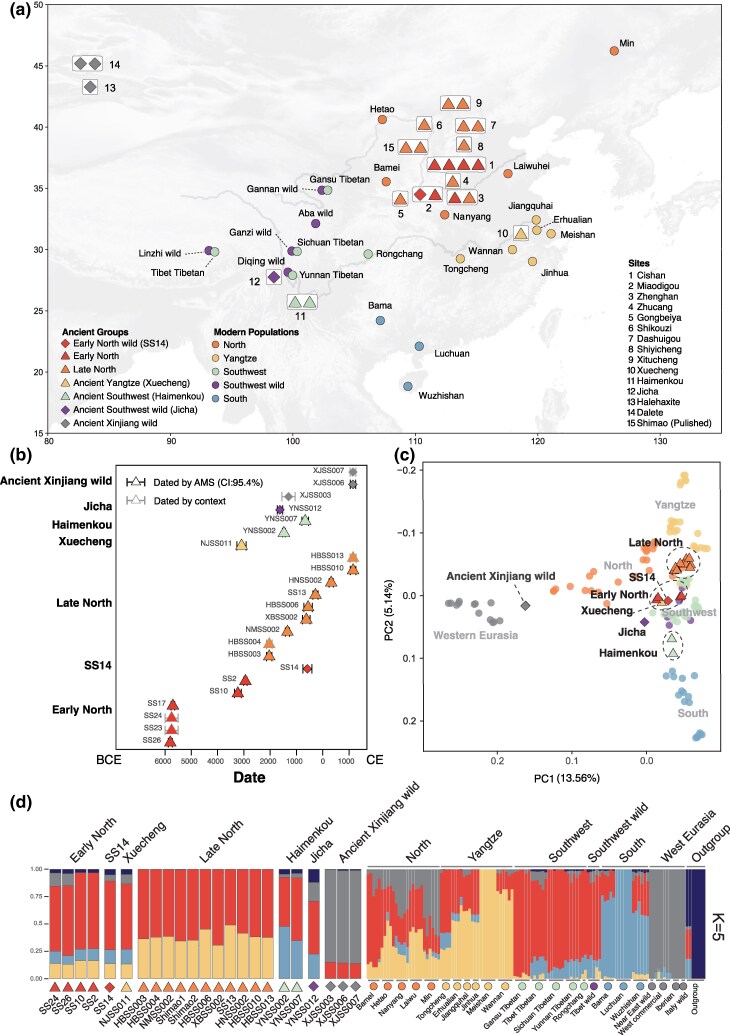
Sample information and population structure of ancient Chinese domestic pigs and wild boar. a) The geographic locations of the studied samples. Triangles, diamonds, and circles represent ancient domestic pigs, ancient wild boar, and published modern populations, respectively. Colors represent the geographical regions or genetic groups. Details of samples are provided in supplementary tables S1a and S1c, Supplementary Material online. b) Ages of ancient samples. The *x*-axis shows the average age, and error bars represent the 95.4% confidence interval (CI) of C^14^ dates, or archaeological time range (supplementary tables S1a and S1b, Supplementary Material online). The colors are the same as panel A. c) PCA of Eurasian pigs and wild boar with ancient samples projected onto the PCs calculated with modern samples. Shapes and colors of the symbols match those in panel A. d) Results of the ADMIXTURE analysis at *K* = 5. The symbols below the bar plot correspond to those in panel A and represent the groups or populations to which each sample or breed belongs. Details of the analysis are provided in supplementary material section S4, Supplementary Material online.

We extracted ancient DNA from 89 samples from 14 archaeological sites across China, constructed 116 sequencing libraries, and generated 21 nuclear genomes (0.1× to 8.6× coverage) and 23 mitochondrial genomes (7.1× to 902.5×). Based on body sizes, population death ages, and archaeological contexts or stable isotope values, 18 samples were classified as domestic pigs and five as wild boars (supplementary tables S1a and S1b, supplementary material section S1, Supplementary Material online). These include 16 individuals from Northern China spanning a 7,000-year period (5,900 BCE to 1,300 CE), one from Lower Yangtze River Basin (∼3,000 BCE), three from Southwestern China (∼1,530 to 540 BCE), and three from Xinjiang (∼1,100 BCE to 1,200 CE) (Figs. 1a and 1b, supplementary table S1a, Supplementary Material online). We analyzed this dataset alongside 2 previously published ancient pig genomes (∼0.2×) from Shimao in Northern China (∼2,000 BCE) (supplementary table S1c, Supplementary Material online) (Wen et al. 2022), and 113 modern genomes from Eurasia. In addition, 7 genomes from *Phacochoerus africanus*, *Sus barbatus*, *Sus verrucosus*, and *Sus scrofa vittatus* were included as outgroups (Fig. 1a, supplementary table S1c, Supplementary Material online) (Frantz et al. 2015; Chen et al. 2020; Zhang et al. 2020). Lastly, we integrated previously published mitogenomes derived from 44 ancient individuals (11.6 to 269.2×) (Wen et al. 2022; Zhang et al. 2022) to assess the mitochondrial variability of domestic pigs and wild boar across East Asia (supplementary table S1d, Supplementary Material online).

## Results

### Local Origin and Genetic Continuity of Domestic Pigs in Northern China

Our mitochondrial phylogenetic and network analysis corroborated previous studies by showing that Chinese domestic pigs generally lacked phylogeographical and temporal structure (supplementary figs. S1 and S2, supplementary material section S2, Supplementary Material online) (Larson et al. 2010; Wen et al. 2022; Zhang et al. 2022). In contrast, the patterns in the nuclear data shown on Principal Component Analysis (PCA) depict a clear geographical structure among the analyzed ancient and modern pigs.

Calculated using 113 modern pigs and wild boar, the first principal component (PC1) separated Eastern and Western Eurasian pig populations (Fig. 1c). Modern breeds in Northern China plotted close to Western Eurasian populations, reflecting their extensive genetic interactions with European pig breeds since the 19th century (Yang et al. 2017; Peng et al. 2022). Among ancient pigs and wild boar, three wild boar from the sites of Dalete and Halehaxite in Xinjiang exhibited close affinity to Western Eurasian *Sus* populations, consistent with their geographic proximity to Central and West Asia (Fig. 1c).

The second principal component (PC2) separated East Asian populations from different regions and time periods. The ancient Northern Chinese samples clustered into 2 temporally distinct groups, hereafter referred to as “Early North” and “Late North”. In the PCA, the four Early North domestic pigs (∼5,900 to 3,000 BCE) were indistinguishable from an ancient wild boar (SS14, ∼500 BCE) in the region, suggesting a local origin from Northern Chinese wild boar. The Late North group consisted of 9 newly reported and 2 previously published ancient pigs (Wen et al. 2022) from 8 archaeological sites (∼2,100 BCE to 1,300 CE) (Figs. 1b and 1c). All of these samples plotted close to modern pigs in the Yangtze River Basin and some Northern Chinese pigs that exhibited less Western Eurasian ancestries on PC2 (Fig. 1d, supplementary table S1c, Supplementary Material online). These patterns were corroborated by the autosomal phylogenetic tree (supplementary fig. S3, Supplementary Material online), and population clustering analyses using ADMIXTURE, Dystruct, and Struct-*f*_4_, all of which revealed a close affinity between Late North pigs and modern pigs from Northern China and the Yangtze River Basin (Fig. 1d, supplementary figs. S4 to S6, Supplementary Material online).

Given the presence of 2 different pig groups in the same region across different time periods, we evaluated whether there was any genetic turnover in Northern China using pairwise outgroup *f*_3_-statistics (supplementary fig. S7, supplementary table S2a, Supplementary Material online) and *f*_4_-statistics (supplementary table S2b, Supplementary Material online). All Late North individuals were closely related to each other, as revealed by the highest *f*_3_ values shared among the members within the group (supplementary fig. S7, supplementary table S2a, Supplementary Material online). We therefore grouped these individuals for subsequent analyses (supplementary fig. S8, supplementary table S2c, Supplementary Material online).

**Fig. 2.**
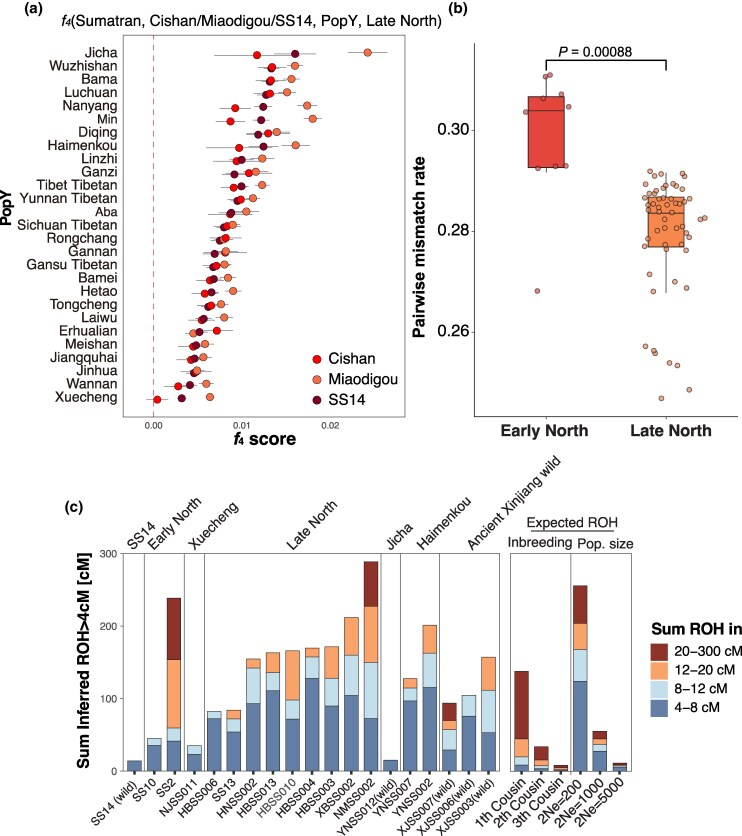
Genetic relationships of ancient northern domestic pigs. a) *F_4_*-statistics in the form *f_4_* (Sumatran, Cishan/Miaodigou/SS14, PopY, Late North). PopY includes all ancient and modern populations analyzed in this study, with the exception of the ancient pigs and wild boars from Northern China. Dots and error bars indicate the *f_4_* values with 3× standard errors (SD) (supplementary table S2d, Supplementary Material online). b) The genetic diversity of Early North and Late North shown by pairwise mismatch rates. The significance between groups was tested with two-tailed t-test. c) ROH segments (>4 cM) detected in ancient genomes. The segments were colored by lengths. Details of the analysis and additional results were provided in supplementary fig. S10, Supplementary Material online. Details of the results are provided in supplementary material section S6, Supplementary Material online.

The Early North individuals also shared the closest genetic affinity with Late North compared with other ancient and modern populations, as suggested by *f_3_* values (supplementary fig. S7, supplementary tables S2a and S2c, Supplementary Material online) and *f*_4_ (Sumatran, Cishan/Miaodigou/SS14, Other populations, Late North) (Fig. 2a and supplementary table S2d, Supplementary Material online). These results suggested that ancient Northern Chinese domestic pigs did not experience a genetic turnover, but formed a genetically continuous population through time, maintaining the same primary ancestral component despite chronological population structure. This continuity was also supported by the lack of temporal pattern in our mitochondrial phylogenetic analysis (supplementary figs. S1 and S2, Supplementary Material online), and was consistent with previous studies (Wen et al. 2022; Zhang et al. 2022).

Notably, the most recent individuals in the Early North group, two ∼5,000-year-old pigs from Miaodigou, exhibited a closer genetic affinity to the Late North pigs after 2,000 BCE than to other Early North members, based on outgroup *f_3_*-statistics and *f_4_* (Sumatran, Late North, Cishan/SS14, Miaodigou) (supplementary figs. S7 and S8, supplementary tables S2c and S2e, Supplementary Material online). This suggests that the Miaodigou pigs, though classified within the Early North group, may represent a transitional stage leading to the emergence of the Late North group.

Collectively, our analyses supported Northern China as an independent center of pig domestication in East Asia. Our analyses also demonstrate genetic continuity in this population spanning approximately 7,000 years, from their initial appearance ∼5,900 BCE until substantial Western Eurasian genetic introgression began to reshape their diversity in the 19th century (Yang et al. 2017; Peng et al. 2022).

### Loss of Genetic Diversity in Northern Chinese Domestic Pigs Led by Intensified Management

By at least 2,100 BCE, domestic pigs in Northern China exhibited genomic differentiation from their wild progenitors, which could have resulted from either gene flow from other regions or the accumulation of genetic drift accentuated by intensified human selection. Given the close affinity between Late North and modern pigs along the Lower Yangtze River revealed by the PCA (Fig. 1c), and the evidence of independent pig domestication in this region, we first tested whether gene flow from the Lower Yangtze River Basin could have contributed to the observed differentiation.

We analyzed the genome of a ∼5,000-year-old Neolithic pig from the Lower Yangtze River Basin (NJSS011, H31-1 at the site of Xuecheng), temporarily situated between the Early and Late North groups (Fig. 1b, supplementary table S1a, Supplementary Material online). The non-significant values of *f*_4_ (Sumatran, Xuecheng, Early North pops, Late North pops) (supplementary table S2b, Supplementary Material online) suggested that the Neolithic domestic pigs from the Yangtze River Basin, or at least those represented by the Xuecheng individual, did not contribute to the transition from Early to Late North pigs in Northern China.

Interestingly, the Xuecheng individual lacked close genetic affinity with local modern populations. Instead, it clustered with Early North individuals, as demonstrated by the PCA (Fig. 1c), and population clustering analysis (Fig. 1d; supplementary fig. S4, Supplementary Material online). This pattern could be explained by gene flow from Northern Chinese pigs as early as 3,000 BCE, or by a shared genetic background between wild boar populations in Northern China and the Yangtze River Basin. Without comparative data from local wild boar, we currently lack the power to distinguish between these possibilities.

We then explored whether the genetic differentiation was associated with genetic drift accumulated during the loss of genetic diversity. By estimating the pairwise mismatch rates within Early North and Late North groups, respectively, we observed significantly lower population diversity in the Late North group (*P*-value <0.01), indicating a decline of genetic diversity (fig. 2b). The more recent individuals also possessed larger amounts of accumulated Runs of Homozygosity (ROH) segments (>4 cM), corresponding to a smaller effective population size (*N_e_*) compared to Early North (Fig. 2c) (Ringbauer et al. 2021). Based on the amount of short ROHs (4 to 8 cM), we estimated a ∼80% reduction in *N_e_* from Early North pigs and wild boar (*N_e_* = 668) to Late North (*N_e_* = 141) (supplementary table S3, Supplementary Material online).

We also identified 2 individuals that possessed long ROH segments (>20 cM) indicating inbreeding, which was likely the result of human-mediated reproduction: SS2 (Miaodigou, ∼3,000 BCE), the most recent individual within the Early North group, and NMSS002 (Shikouzi, ∼1,500 BCE) from the Late North group (Fig. 2c, supplementary fig. S9, Supplementary Material online). The time of this genetic diversity decline and recent inbreeding appeared to be correlated with the intensified pig management, which, as suggested by zooarchaeological evidence, may have begun as early as 3,000 BCE (Dong and Yuan 2020; Zhang et al. 2021; Yang et al. 2022; You et al. 2024).

### Ancient Southwestern Pigs Were Derived from Northern Pigs

Genetic studies of modern pigs suggested that Southwestern China was an independent domestication center (Peng et al. 2022), while archaeological evidence indicated that agricultural practices were introduced from Northern China to Southwestern China by millet farmers between 3,400 and 3,000 BCE (Driscoll et al. 2007; Liu et al. 2022; Tao et al. 2023). To test this hypothesis of an independent center, we analyzed the genomic data from 2 ancient domestic pigs (Haimenkou, ∼1,500 to 700 BCE) and one wild boar (Jicha, 1,700 to 1,500 BCE) from Southwestern China (Figs. 1a and 1b, supplementary table S1a, Supplementary Material online).

The ancient domestic pigs (Haimenkou) from the region were closely related to modern pigs from Southwestern and Southern China based on PCA and an autosomal phylogenetic tree (Fig. 1c, supplementary fig. S3, Supplementary Material online). Intriguingly, the Jicha wild boar was basal to all Eurasian *Sus scrofa* in the phylogenetic tree (supplementary fig. S3, Supplementary Material online). This finding was further supported by outgroup *f_3_*-statistics, in which the Jicha individual shared the lowest genetic affinity with all other Eurasian groups, while Haimenkou showed greater affinity to the ancient pigs from Northern China, and modern pigs from Southwestern and Southern China (supplementary figs. S7 and S8, supplementary tables S2a and S2c, Supplementary Material online). The genetic distinctiveness between the ancient domestic pigs and the wild boar suggested that the pigs were not domesticated from local boar, but were instead introduced from Northern China, as initially suggested by the archaeological evidence (Driscoll et al. 2007; Liu et al. 2022; Tao et al. 2023).

**Fig. 3.**
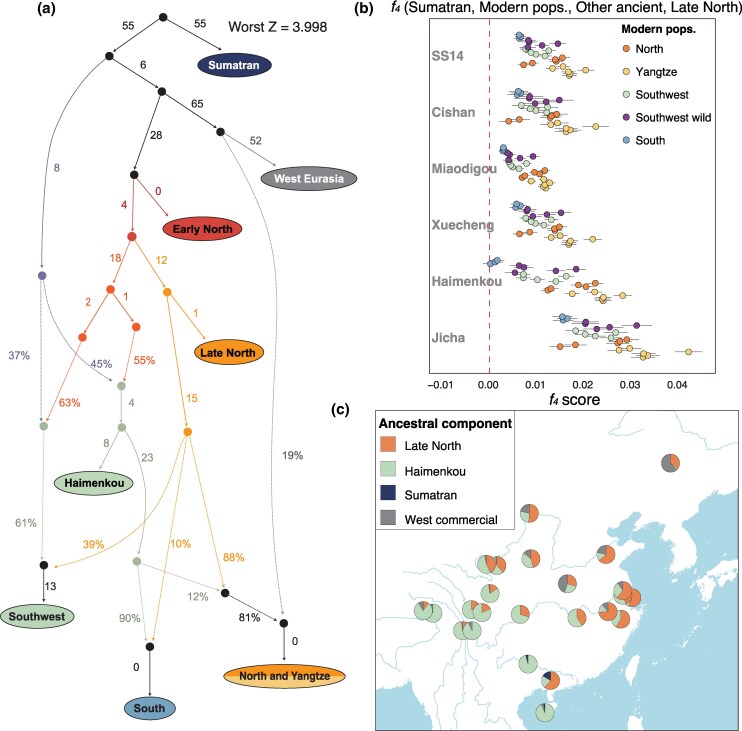
The genetic composition of modern Chinese pigs. a) Admixture graph modeling of ancient and modern populations using qpGraph. Details of the modeling are described in supplementary material section S7, Supplementary Material online. b) *F_4_*-statistics in the form *f_4_* (Sumatran, Modern pops, Other ancients, Late North). Colors of dots denote geographic regions of each modern population as described in Fig. 1. The dots and error bars show the *f_4_* values with 3× SD (supplementary table S2g, Supplementary Material online). c) QpAdm modeling of modern populations, with Late North, Haimenkou, Sumatran and West commercial representing ancient Northern pigs, ancient Southwestern pigs, undescribed Southern wild boars, and Western Eurasia pigs. Pie charts show the estimated proportion of each ancestry. Results using Miaodigou as a replacement for Late North are shown in supplementary fig. S11, supplementary tables S4b and S4c, Supplementary Material online.

To test whether the pigs introduced from Northern China interbred with local wild boar in Southwestern China, we applied *f_4_* (Sumatran, Jicha; Haimenkou, Miaodigou/Late North) analysis (supplementary table S2f, Supplementary Material online). We did not detect any significant genetic contribution from the Jicha wild boar. A qpGraph analysis, however, suggested that the Haimenkou pigs were a mixture of a sister-lineage of the Late North population and an unsampled lineage basal to Eurasian pigs (Fig. 3a, supplementary fig. S13, Supplementary Material online). These analyses revealed that the domestic pigs in Southwestern China, dated to at least ∼1,500 BCE, were genetically distinct from local wild boar, and closely associated with ancient Northern pigs, suggesting that millet farmers from Northern China brought their pigs to Southwestern China (Tao et al. 2023).

### Genetic Composition of Modern Chinese Domestic Pigs

We then investigated whether separate ancient domestic pig populations contributed differently to the genetic diversity of modern Chinese pigs. The PCA (Fig. 1c), autosomal phylogenetic tree (supplementary fig. S3, Supplementary Material online) and outgroup *f_3_*-statistics (supplementary fig. S8, supplementary table S2c, Supplementary Material online) revealed clear genetic structure among modern breeds. Breeds from Northern China and the Yangtze River Basin showed close affinity to the Late North population, whereas those from Southwestern and Southern China were more closely related to the ancient Southwestern pigs. The *f*_4_-statistics in the form *f*_4_ (Sumatran, Modern pops, Other ancient, Late North) further revealed that, relative to all other ancient pig populations, most modern pig breeds shared a closer affinity to Late North pigs (Fig. 3b, supplementary table S2g, Supplementary Material online).

Notably, several breeds from Southern China that possessed comparable affinity with both Haimenkou and Late North pigs, deviated from this overall pattern (Fig. 3b, supplementary table S2g, Supplementary Material online). These findings demonstrated that ancient Northern pigs had a widespread genetic influence on the formation of modern pig populations across China.

To quantify the ancient Northern pig contribution and to assess whether admixture among ancient pigs contributed to the genetic structure of modern pigs, we modeled the ancestral composition of modern pigs from different regions using qpAdm analysis. This analysis revealed that most of the modern pigs could be modeled as an admixture of ancient Northern pigs (represented by group Late North or Miaodigou), Southwestern pigs (represented by Haimenkou), and commercial breeds from Western Eurasia (Fig. 3c, supplementary fig. S11, supplementary tables S4a, S4b and S4c, Supplementary Material online). Ancient Northern pig ancestry was detected in all modern Chinese breeds, and those from Northern China and the Yangtze River Basin possessed the highest proportions up to 86.4% (Fig. 3c, supplementary fig. S11, supplementary tables S4a, S4b and S4c, Supplementary Material online). This was consistent with the *f_4_*-statistics that showed that all modern breeds in the 2 regions shared the highest genetic affinity with the Late North group (Fig. 3b, supplementary table S2g, Supplementary Material online). These findings highlighted the extensive dispersal of Northern pigs into the Lower Yangtze River Basin, where they likely replaced the earlier population represented by the Xuecheng individual.

Additionally, we identified recent and substantial introgression from European domestic pigs into Northern Chinese pigs. The proportion of European ancestry in Northern breeds was estimated to be 14.4% to 61.5% (Fig. 3c, supplementary fig. S11, supplementary tables S4a and S4b, Supplementary Material online). This introgression also explained the reduced genetic affinity of Northern pigs to their ancient counterparts, compared to modern populations from the Yangtze River Basin, which have been minimally influenced by the European introgression (Yang et al. 2017; Wen et al. 2022).

The modern pigs from Southwestern China were modeled as having derived 58.1% to 88.5% of their genomic ancestry from the Haimenkou pigs. The remaining proportion was attributed to ancient Northern pigs (represented by Late North in Fig. 3c, supplementary tables S4b and S4c, Supplementary Material online, or Miaodigou in supplementary fig. S11, supplementary tables S4a and S4c, Supplementary Material online). Together with their closer affinity to Late North than Haimenkou pigs revealed by *f_4_* (Sumatran, Southwestern modern pops; Haimenkou, Late North) (Fig. 3b, supplementary table S2g, Supplementary Material online), this result suggested that modern local breeds have acquired additional Northern ancestry after the formation of the Haimankou population, indicating a persistent influence of Northern pigs in Southwestern populations.

Haimenkou ancestry was also detected in modern pigs across Northern China and the Yangtze River Basin, with the highest proportions observed in breeds from the Upper or Middle Yellow (e.g. Bamei) and Yangtze River (e.g. Tongcheng) Basins. This pattern suggested a bidirectional diffusion of ancestries between Northern/Eastern and Southern/Western China (Fig. 3c, supplementary fig. S11, supplementary tables S4a, S4b and S4c, Supplementary Material online). However, we did not detect the additional Haimenkou affinity in Late North individuals dated to as recently as ∼1,300 CE (represented by the Xitucheng pigs) using *f*_4_ (Sumatran, Haimenkou; Late North site 1, Late North site 2) (supplementary table S2b, Supplementary Material online). These findings suggested that the observed diffusion of Southwestern ancestry occurred only in the last few centuries.

Modern wild boar from the Qinghai-Tibet Plateau (labeled as Ganzi, Diqing, Gannan, Linzhi, and Aba) (Li et al. 2013) also possessed genetic profiles similar to modern and ancient local pigs (Haimenkou and Tibetan breeds), but distinct from the ancient local wild boar (Jicha, YNSS012) (Figs. 1a, 1d, 3b and supplementary table S1c, Supplementary Material online). This suggests that the ancient dispersal of Northern ancestries may have reshaped modern wild boar populations, which raises an extra concern regarding future genetic studies of wild boar since their genomes may have been influenced by admixture with domestic pigs.

All 3 breeds in Southern China possessed predominantly Haimenkou ancestry, which supports a genetic link between Southern and Southwestern Chinese pigs. Southern Chinese pigs also possessed ∼5% ancestry, best represented by Sumatran wild boar (Fig. 3c, supplementary fig. S11, supplementary table S4c, Supplementary Material online). These results suggest that, in addition to the ancient Southwestern pigs, an undescribed, indigenous lineage closely related to Southeast Asian wild boars also contributed to pigs in Southern China.

To comprehensively understand how the dispersal and subsequent gene flow have shaped modern pig populations, we further used qpGraph to model this process. We found that the Early North group was basal to all the East Asian lineages. One lineage gave rise to Late North, and then to the modern North and Yangtze pigs, after mixing with Haimenkou-related and Western Eurasian-related lineages. The other lineage mixed with a ghost lineage basal to all Eurasian *Sus scrofa* and formed Haimenkou pigs as well as modern Southwestern and Southern Chinese pigs. Modern Southwestern and Southern pigs also received additional gene flow from the Late North-related lineage (Fig. 3a, supplementary figs. S12 to S17, Supplementary Material online).

### Temporal and Geographical Origins of the Black Coloration in Chinese Domestic Pigs

Previous studies have demonstrated that all modern Chinese indigenous pigs possess a dominant black coat phenotype, caused by a single nonsynonymous mutation (L102P Chr6:181,883) in the *MC1R* (Fang et al. 2009; Linderholm et al. 2016). To assess when and where this mutation first arose, we tested its presence in ancient Chinese domestic and wild individuals. We found that eight domestic pigs and four wild boar (out of 20 ancient domestic pigs and 5 ancient wild boar) possessed sequencing reads spanning the L102P SNP (supplementary table S5a, Supplementary Material online). The 8 ancient domestic pigs all had at least 1 read carrying the causative allele for black coat, including the oldest individuals from Miaodigou in Northern China, dating to ∼3,000 BCE (Fig. 1b, supplementary table S1a, Supplementary Material online). The wild boar in Northern China (SS14; 500 BCE) and Xinjiang (XJSS003, XJSS006, and XJSS007; 1,000 BCE to 1,500 CE), only possessed reads carrying the ancestral, camouflage-coding alleles (supplementary table S5a, Supplementary Material online).

**Fig. 4.**
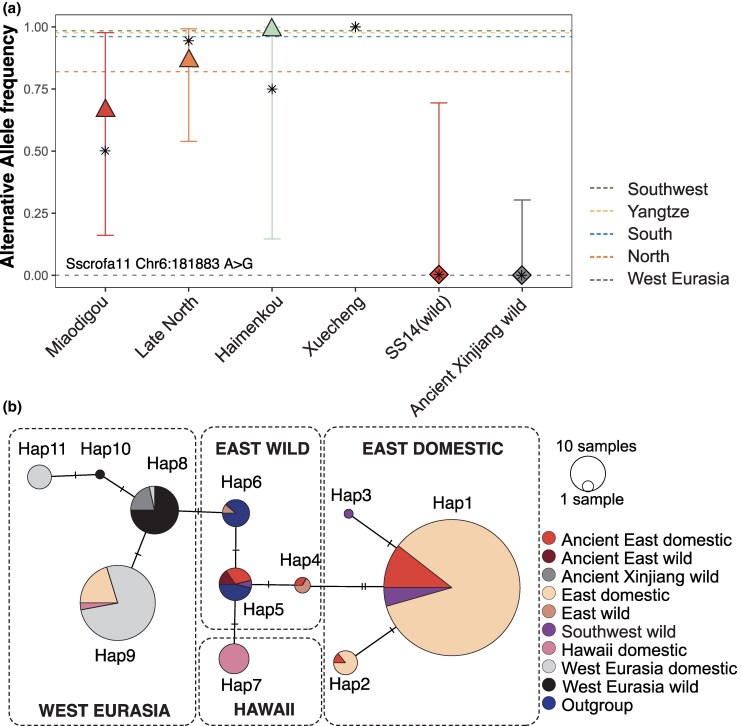
The origin of black coloration in Chinese domestic pigs. a) Frequency of the alternative allele in the *MC1R* gene (Sscrofa11 Chr6:181,883 A > G) associated with black coloration in ancient domestic pigs and wild boars. Triangles (domestic), diamonds (wild), and error bars indicate maximum likelihood (ML) estimates and 95% CI intervals based on mapped reads, and asterisks indicate allele frequencies directly calculated from imputed data (supplementary fig. S18, supplementary tables S5b and S5c, Supplementary Material online). Xuecheng, which did not have reads covering the target site, was only shown with the allele frequency from imputed data. The dashed lines in different colors represented the allele frequencies calculated from various modern domestic pig populations using imputed data. b) Network constructed using the imputed and phased haplotype of the *MC1R* gene (Sscrofa11, Chr6:181,225 to 182,187). Details of the analysis and haplotype assignments are provided in supplementary material section S8 and supplementary tables S5d and S5e, Supplementary Material online.

We then estimated the allele frequencies in ancient and modern populations using a maximum likelihood (ML)-based algorithm (Fig. 4a, supplementary fig. S18, supplementary tables S5b and S5c, Supplementary Material online) (Mathieson et al. 2015). In modern Chinese domestic pigs, the causative mutation was nearly fixed, except in some Northern Chinese breeds, where ∼18% of individuals possessed non-East Asia haplotypes in their *MC1R* genes, possibly due to gene flow from Western Eurasian pigs.

In ancient Chinese pigs, we observed an increase in the causative allele frequency from Early North pigs ∼3,000 BCE (Miaodigou, *n* = 2, 66.7%, 95% CI: 16.1% to 97.7%) to Late North pigs more recent than 2,000 BCE (Late North, *n* = 5, 87.0%, 95% CI: 53.9% to 99.2%) (Fig. 4a, supplementary table S5b, Supplementary Material online). By contrast, modern Western Eurasian populations and ancient wild boars from China carried the East Asian causative allele at a frequency less than 5% (Fig. 4a, supplementary table S5b, Supplementary Material online), highlighting the absence of this East Asian-specific variant outside Chinese domestic pigs.

To expand our dataset and increase resolution, we imputed and phased 17 newly reported and 2 published ancient genomes (Wen et al. 2022) with depth >0.3×, together with all modern genomes on chromosome 6, to reconstruct the *MC1R* haplotypes (Fig. 4b, supplementary table S5d, Supplementary Material online). After merging our data with 96 published *MC1R* gene sequences (Linderholm et al. 2016), we performed haplotype network analysis and observed a clear haplotype distinction among domestic pigs and wild boars in Eastern and Western Eurasia (Fig. 4b, supplementary figs. S19 and S20, supplementary tables S5d and S5e, Supplementary Material online).

All ancient and 96.8% modern Chinese domestic pigs possessed at least one copy of the three East domestic haplotypes (Hap1–Hap3), which carried the causative allele for black coloration (Fig. 4b, supplementary tables S5e, Supplementary Material online). Although we were unable to genotype the oldest Cishan pigs (5,900 BCE) due to low sequencing depth, we found that the East Asian black haplotype can be traced back at least to the 3,300 to 3,000 BCE Miaodigou pigs (SS2 and SS10) from Northern China, and the 3,000 BCE Xuecheng individual from the Yangtze River Basin (NJSS011) (Fig. 4b, supplementary tables S5d and S5e, Supplementary Material online). Furthermore, with the imputed genotypes, we recalculated the allele frequencies of different populations (Fig. 4a, supplementary tables S5b and S5c, Supplementary Material online) and confirmed the increase of causative allele frequency from Early to Late Northern pigs. This result indicated that the black coat was presumably subject to ongoing selection before it became fixed.

We found that the wild boar haplotype carried by ancient Chinese pigs and the 2,500-year-old wild boar from Northern China (SS14) was the same as the Sumatran wild boar haplotype (Hap5), indicating that it was common in East Asian wild boar populations at this time. The three ancient Xinjiang wild boar shared the same haplotype with Italian and Near Eastern wild boars (Hap8), which corresponds to their close affinity with Western Eurasian pigs and wild boar shown on the PCA (Fig. 1c). Ten modern domestic pigs from Northern China carried the European domestic pig haplotype (Hap9), which is consistent with recent gene flow from European commercial breeds in this region (Wen et al. 2022). Interestingly, all the Tibetan (Southwest) wild boars carried at least one copy of the Asian domestic haplotypes (Hap 1 and 3), further suggesting genetic introgression from domestic pigs (Fig. 4b, supplementary fig. S20, supplementary tables S5d and S5e, Supplementary Material online). A similar phenomenon has also been observed in Europe, where the domestic haplotypes in the *MC1R* gene were frequently detected in local wild boars and used as a marker of the introgression from domestic pigs (Fontanesi et al. 2014; Canu et al. 2016). Our results suggested that this method could also be applied to evaluate gene flow from domestic pigs into local wild boar in China.

Based on these findings, we traced the origin of the black coat phenotype in East Asian domestic pigs to Northern China and the Lower Yangtze River regions. Limited by the low genome coverages of the ∼5,900 BCE Cishan pigs (supplementary tables S1a, S5a and S5d, Supplementary Material online), we were unable to identify their genotypes. Therefore, the oldest high-coverage individuals in our dataset, the Miaodigou pigs, were the earliest known individuals carrying the causative mutation for black coloration (supplementary tables S5a and S5e, Supplementary Material online), and the 3,000 BCE Xuecheng pig was the earliest individual homozygous for the East domestic haplotype (supplementary tables S5a and S5e, Supplementary Material online).

Considering the uncertain origin of the Yangtze individual, which could potentially be linked to Northern pigs, and the long history of pig domestication in Northern China, we suggest that the black coloration was more likely to have originated in Northern China. The genotype was likely selected and became fixed in Northern pig populations after 2,000 BCE (Fig. 4a, supplementary table S5c, Supplementary Material online). Given their substantial genetic contribution to most Chinese pig populations, we suggest that the spread of this black coat color-related genotype throughout China was also related to the dispersal of Northern domestic pigs.

## Discussion

Overall, our results demonstrate that the earliest domestic pigs in Northern China, from ∼5,900 BCE were genetically similar to local wild boar, thus supporting the initial domestication of pigs in Northern China (Fig. 1). On the contrary, the genetic affinity between early domestic pigs in Southwestern China and ancient Northern Chinese pigs, together with their distinction with local wild boar, suggested that Southwestern China was not an independent center of domestication.

Around or soon after 3,000 BCE, the early domestic population in Northern China became genetically differentiated from their wild progenitors, likely as a result of genetic drift associated with bottlenecks, which also led to an overall reduction in genetic diversity and recent inbreeding as early as ∼3,000 BCE (Figs. 1b and 2c). Intriguingly, zooarchaeological and stable isotopic studies indicate a shift in pig management practices from extensive to an intensive strategy between 4,000 and 3,000 BCE (Dong and Yuan 2020; Zhang et al. 2021; Yang et al. 2022; You et al. 2024), which coincides with the genomic ancestry transition from Early to Late North pigs. The intensified breeding practice, involving a homogeneous living environment and possibly stronger human-mediated selection, reduced genetic diversity and led to recent inbreeding as a result of human-controlled reproduction (Zeder 2012; Frantz et al. 2020).

A traditional view of domestication holds that founder effects during the early stages of domestication led to low genetic diversity in modern domestic animals, with cattle being the most prominent case (Rossi et al. 2024). However, it has been challenged by recent studies on ancient domestic animals, such as dogs (Larson et al. 2012), chickens (Loog et al. 2017), and horses (Librado et al. 2024), which showed that the genetic diversity was mainly lost during the past few centuries as a result of more intensive selection. Our study suggests that in East Asian pigs, the major loss of genetic diversity occurred neither during the initial stages of domestication, nor in recent centuries, but rather in association with changes in management ∼3,000 years after domestication, highlighting the need for future studies to elucidate the underlying mechanisms.

**Fig. 5.**
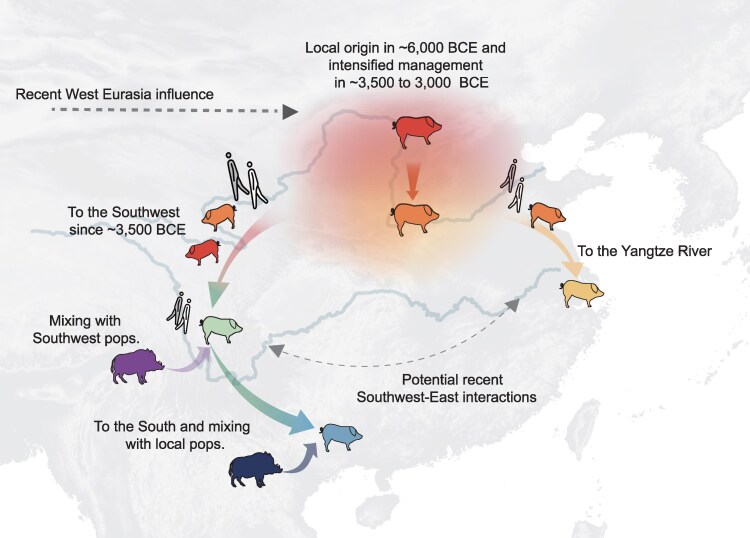
Illustration of the domestication history of Chinese pigs. The arrows show the genetic transition related to the changes in management practices, dispersal along the human migration routes, and interactions with local wild boars. The colors of different populations of domestic pigs and wild boars are consistent with Fig. 1. A detailed description of this figure is provided in supplementary material section S9, Supplementary Material online.

Following their domestication, pigs with black coats from Northern China dispersed across East Asia and formed the majority of the modern pig gene pool across China. Their expansion may have followed 2 major routes, both closely mirroring human dispersals (Fig. 5). Pigs traveled southeast and arrived in the Yangtze River Basin, where they contributed to the formation of modern local breeds. A genetic study on ancient East Asian people also described the influence of Northern Chinese people in Southern populations since the Late Neolithic (Yang et al. 2020).

The arrival of pigs in Southwestern China likely accompanied the migration of Yellow River millet farmers, who reached the edge of the Qinghai-Tibet Plateau ∼5,400 to 5,000 years ago (Wang et al. 2023), and Southwestern China before 3,000 years ago (Tao et al. 2023). These pigs also admixed with local wild boar, consistent with the detection of Southeast Asian-related Hòabìnhian ancestry in Southwestern Chinese farmers (Tao et al. 2023).

In addition to the initial dispersal of Northern pigs, a bidirectional diffusion of genetic ancestries between pigs from the Southwest and North and Yangtze River Basin was observed in the genomes of modern breeds in these regions (Fig. 5). Both dispersal waves contributed to pig populations in Southern China, mirroring the observation of Northern and Southern East Asian ancestries found in historical Southern Chinese human populations (T. Wang et al. 2021b). The lack of ancient genomic data prevents us from determining which of the ancestries arrived in this region first, and whether the Southeast Asian-related ancestry found in this region represented local wild boar or an independently domestic population that was later largely replaced. Further ancient genomic studies are necessary for understanding the formation of pig populations and the associated patterns of human migration in this region.

The recent diffusion among different pig populations is eroding the genetic structure of East Asian pigs that formed over the past millennia. More intriguingly, the exchange of Chinese and European pigs through modern breeding practices is also gradually diminishing the genetic distinction between Eastern and Western Eurasian pigs, which arose from the independent domestication of 2 highly divergent wild boar populations that had been isolated for more than a million years (White 2011; Yang et al. 2017; Wen et al. 2022).

This process represents a clear case of biotic homogenization (Olden et al. 2004), which may have already eliminated unique genetic resources in East Asian domestic pigs. This is especially true for Northern Chinese pigs, which maintained genetic continuity for approximately 7,000 years prior to this admixture. Our study also draws attention to the trend of global genetic integration of domestic pigs. Further ancient genomic research will help to elucidate the genetic and functional consequences of this ongoing process.

## Materials and Methods

### Sampling

All the specimens newly collected for this study came from archaeological excavations and were obtained through collaboration with the excavators or zooarchaeologists working on these samples. Each sample has a well-documented provenance of the archaeological remains or stratigraphic layers from which it was retrieved, ensuring the accuracy of its archaeological context. Details about the archaeological contexts for those samples are provided in supplementary material section S1 and supplementary tables S1a and S1b, Supplementary Material online.

### Radiocarbon Dating

We report 14 new direct accelerator mass spectrometry (AMS) radiocarbon dates from ancient samples obtained from Beta Analytic, USA, and from the School of Archaeology, Peking University, China. Other samples were dated indirectly using charcoal or bones from the same stratigraphic layer, or inferred based on their archaeological contexts. Details of the dating information are provided in supplementary tables S1a and S1b, Supplementary Material online. All radiocarbon dates reported were calibrated using the INTCAL20 calibration curve(Reimer et al. 2020).

### Ancient DNA Laboratory Work

All the samples were processed in the dedicated ancient DNA laboratory at the School of Life Sciences, Peking University. DNA was extracted following the protocol published by Dabney et al. (Dabney et al. 2013) with slight modification, including a pre-digestion step or sodium hypochlorite treatment (Boessenkool et al. 2017). Libraries were built using the Blunt-End Single-Tube (BEST) double-stranded library building method (Carøe et al. 2018) or the Santa Cruz Reaction (SCR) single-stranded library construction method (Kapp et al. 2021). For double-stranded libraries, some samples were processed with the “Half-UDG” treatment to remove most of the cytosine deamination damage (Briggs et al. 2010; Rohland et al. 2015). All libraries were purified using Solid-Phase Reversible Immobization beads (Beckman Coulter) and eluted in 40 μL of Elution Buffer. Libraries were subsequently double-indexed using standard Illumina P5 and P7 primers (supplementary tables S1a and S1b, Supplementary Material online).

All libraries were screened on an Illumina NovaSeq 6,000 platform (Modi et al. 2021) using paired-end 150 bp (PE150) sequencing at Novogene, Beijing. Based on the screening results, selected samples underwent deeper sequencing on the MGI Tech MGISEQ-2000 (Korostin et al. 2020) sequencer using paired-end 100-bp (PE100) strategy at the National Centre for Protein Sciences, Peking University (supplementary tables S1a and S1b, Supplementary Material online).

## Quantification and Statistical Analysis

### Sequencing Data Processing

We processed ancient sequencing data using the nf-core framework-based EAGER v2.4.4 pipeline (Ewels et al. 2020; Yates et al. 2021) to perform quality control and alignment of sequencing data. Published ancient sequencing data were first converted from NCBI Sequence Read Archive (SRA) format to fastq.gz format using “fastq-dump” from Sratoolkit v3.0.0 (https://hpc.nih.gov/apps/sratoolkit.html) and jointly aligned with the data newly generated in this study. Within the workflow, the quality control of sequencing reads was performed using FastQC v0.11.9 (Andrews 2010). Adapter sequences were removed using AdapterRemoval v2.3.2 (Schubert et al. 2016), with the “–mergedonly” option to keep only merged reads for downstream mapping. The merged reads were aligned to the domestic pig reference genome (Sscrofa11.1 NCBI: GCF_000003025.6) using BWA v0.7.17 (aln/samse) (Li and Durbin 2009). Endogenous DNA content was calculated using a custom Python script based on SAMtools v1.12 (Li et al. 2009; Danecek et al. 2021) flagstat results. PCR duplicates were removed using Picard v2.26.0 toolkit (MarkDuplicates). Damage patterns were assessed using DamageProfiler v0.4.9 (Neukamm et al. 2021). Quality control of the deduplicated BAM files was performed using Qualimap v2.2.2 (Okonechnikov et al. 2016). Library complexity was predicted using preseq v3.1 (Daley and Smith 2013). Summary results were generated using MultiQC v1.12 (Ewels et al. 2016). The authenticity of ancient libraries was assessed based on the damage pattern through mapdamage v2.2.1 (Jónsson et al. 2013) and fragment length.

For mitochondrial genome reconstruction, merged reads were mapped to the pig mitochondrial reference genome (NCBI: NC_000845.1) using BWA (aln) and realigned using CircularMapper v1.93.5 (Yates et al. 2021). PCR duplicates were removed using DeDup (Yates et al. 2021), and the deduplicated reads were filtered for a minimum mapping quality of 30 using SAMtools “view” (-q 30). Mitochondrial consensus sequences were generated using Schmutzi endoCaller (Renaud et al. 2015) with a quality threshold of 30 (–qual 30).

We also downloaded 120 published modern genomes for comparison, including 113 domestic pigs and wild boars distributed across the Eurasian continent, and 7 outgroup individuals from other species within *Sus* genus (supplementary table S1c, Supplementary Material online). The downloaded SRA binary files were converted to fastq.gz format using “fastq-dump” from Sratoolkit v3.0.0. Sequencing data from the same individual were merged. Adapter sequences were removed using AdapterRemoval software with “–trimns”, “–trimqualities”, and “–collapse”. After that, both the merged reads (collapsed reads) and unmerged pairwise reads (Pair1 truncated and Pair2 truncated reads), were aligned to the same domestic pig reference genome as ancient samples using BWA (mem) (Li 2013) with the “-M” flag. The mapped reads were sorted and deduplicated using SAMtools markdup.

Modern mitochondrial reads were mapped to the Meishan pig mitochondrial reference genome (GenBank: JN601070.1) using BWA (mem), and sorted and filtered using the same parameters as for nuclear genomes. Consensus mitochondrial sequences of modern samples were obtained using the “consensus” function of SAMtools.

### Genotyping

We identified single-nucleotide polymorphisms (SNPs) based on the 113 published modern samples, using the BCFtools v1.15.1 mpileup (Danecek et al. 2021) with the following parameters: “-Ou -B -C50 -d 70 -q 30 -Q 30 –skip-any-set SECONDARY -I -f”. Variant calling was performed by BCFtools call with “-Ou -A -m -v”. The generated dataset was filtered using BCFtools and Plink v1.9 (Purcell et al. 2007) to exclude transitions to avoid the impact of deamination-induced damage in ancient genome; remove variants with a minor allele frequency smaller than 0.01 (–maf 0.01); exclude variants with a genotyping rate less than 0.2; prune variants using a 10-kb window, step of 2 -SNP step size, and an *R*^2^ threshold of 0.5 (–indep-pairwise 10 kb 2 0.5); remove SNPs deviating from Hardy-Weinberg equilibrium with a *P*-value below 0.0001; retain only biallelic SNPs (–biallelic-only). Finally, we kept a total of 1,261,811 autosomal SNPs (hereafter called 1,262 K). To minimize reference bias, pseudo-haploid genotypes (–randomHaploid) were called for 21 newly reported and 2 published ancient Chinese pig samples on the 1,262 K SNP dataset with SAMtools mpileup and pileupCaller v1.5.2 (Lazaridis et al. 2016) using only cleaned reads with base and mapping quality over 30 (-q 30 -Q 30). The resulting data were merged with modern samples for downstream analysis.

### Mitochondrial Genome Analysis

In this analysis, we used the 23 newly reported ancient specimens, 44 ancient Chinese domestic pigs previously published in other studies (Wen et al. 2022; Zhang et al. 2022), and 113 modern Eurasian domestic pigs and wild boars. Sumatran wild boars were used as the outgroup (supplementary table S1d, Supplementary Material online). Sequences of these samples were aligned using MUSCLE v5.1 (Edgar 2022) with default parameters. The aligned sequences were trimmed using TrimAL v1.4 software (Capella-Gutiérrez et al. 2009) to remove the regions with excessive gaps or ambiguous sites (Ns). A maximum likelihood (ML) phylogenetic tree was constructed using IQ-TREE v1.6.12 (Nguyen et al. 2015), with 1,000 bootstrap replicates. The resulting phylogenetic tree was visualized using FigTree v1.4.4 (http://tree.bio.ed.ac.uk/software/figtree/) and the iTol web tool (https://itol.embl.de/) (supplementary fig. S1, Supplementary Material online). The aligned mitochondrial sequences were also analyzed with DnaSP v6.12.02 (Rozas et al. 2017) to identify haplotypes and estimate their frequencies within each group. A Median-Joining haplotype network was constructed using Arlequin v3.5.2.2 (Excoffier and Lischer 2010) and PopART v1.7 (Leigh et al. 2015) (supplementary fig. S2, Supplementary Material online). Details of the mitochondrial genome analysis are provided in supplementary material section S2, Supplementary Material online.

### Population Genetic Analysis

Principal component analysis (PCA) was performed using SmartPCA v16000 in EIGENSOFT v7.2.1 (Patterson et al. 2006; Price et al. 2006), based on the 1262 K SNP set and calculated from 113 modern Eurasian pigs and wild boars. Ancient samples were projected onto the PC space with “LsqProject: Yes” parameter (Fig. 1c).

An autosomal Neighbor-Joining (NJ) phylogenetic tree was constructed by calculating pairwise genetic distances using the *P*-distance method implemented in a custom script. For bootstrapping, the genotype dataset (1,262 K) was divided into 1,000 segments. A random subset of 300 segments was selected and merged in each of 100 bootstrap replicates. Distance matrices were generated and used to construct the NJ tree with Phylip v3.697 (Retief 1999), followed by consensus tree generation using the consensus module. Phylogenetic trees were visualized using the iTol web tool (https://itol.embl.de/) (supplementary fig. S3, Supplementary Material online). Details of the autosomal NJ tree analysis are provided in supplementary material section S3, Supplementary Material online.

Unsupervised population clustering analysis was performed using ADMIXTURE v1.3.0 (Alexander et al. 2009) on 113 modern Eurasian pigs, 23 ancient samples, and 7 outgroup individuals. A convergence threshold was set to 0.0001 (-c 0.0001). The optimal number of clusters (K) was determined via 10-fold cross-validation (CV) error (–cv = 10) across K values ranging from 2 to 7. *K* = 5 was selected based on the CV error (Fig. 1d, supplementary fig. S4, Supplementary Material online). We also applied Dystruct (Joseph and Pe’er 2019) and Struct-*f_4_* (Librado and Orlando 2022) analyses. In Dystruct, we tested 5 to 7 populations (–npops 5 to 7) across 1,261,811 loci (–nloci 1,261,811). Generation time was set to 2 years, and 10% of loci were randomly held out for validation (–hold-out-fraction 0.1). A random seed of 1,145 (–seed 1,145) was used for reproducibility, and a separate seed of 55,307 (–hold-out-seed 55,307) was used for hold-out sampling. Results are shown in supplementary fig. S5, Supplementary Material online. In Struct-*f_4_*, we modeled 5 to 7 populations (-K 5 to 7), with a maximum of 700,000 *f*_4_-statistics (-m 700,000). Results of the analysis are shown in supplementary fig. S6, Supplementary Material online. Details about the results of unsupervised population clustering are provided in supplementary material section S4, Supplementary Material online.

Outgroup *f_3_*-statistics analysis was calculated using qp3Pop in AdmixTools v7.0 (Patterson et al. 2006, 2012), with Sumatran wild boars as the outgroup. Similarly, the *f*_4_-statistics analysis was computed using qpDstat in AdmixTools v.7.0, enabling “f4mode: Yes,” and Sumatran wild boars as the outgroup. When testing gene flow between Sumatran wild boars and Haimenkou pigs, *Sus barbatus* was used as an alternative outgroup. Both the *f_3_*-statistics and *f_4_*-statistics were applied to ancient archaeological sites to test the grouping of ancient samples (supplementary fig. S7, supplementary tables S2a and S2b, Supplementary Material online) and all populations after the grouping of ancient samples (supplementary fig. S8, supplementary tables S2c, S2d to S2g, Supplementary Material online). Additional details are provided in supplementary material section S5, Supplementary Material online.

### Population Diversity and Runs of Homozygosity (ROH)

To evaluate the genetic diversity of Early North and Late North groups, we calculated pairwise mismatch rates within each group based on the 1,262 K SNPs set with a custom Python script.

ROH segments were identified using hapROH v0.6473 (Ringbauer et al. 2021) for ancient samples with nuclear genome depths above 0.3× (with genomes of SS24 and SS24 from Cishan excluded) (supplementary table S1a, Supplementary Material online). A reference panel was constructed from the SWIM haplotype dataset (Ding et al. 2023) (1,241 modern individuals, 34.6 million SNPs). After intersecting with the 1,262 K SNPs set, 1,043,005 SNPs were retained and formatted in hdf5 with a 1 cM/1 Mb linkage map. ROH segments were binned into 4 length categories (4 to 8 cM, 8 to 12 cM, 12 to 20 cM, > 20 cM) for statistical analysis (Fig. 2c, supplementary fig. S10, Supplementary Material online). All modern samples were analyzed using both hapROH and PLINK v1.9 –homozyg (supplementary fig. S10, Supplementary Material online). For hapROH, we applied parameters consistent with ancient samples, except for e_model=“diploid_gt” to reflect diploid genotype (supplementary fig. S10, Supplementary Material online). Details about the results of these analyses are provided in supplementary material section S6, Supplementary Material online.

We also adopted hapROH to infer the effective population sizes of Early North and Late North groups. Individuals SS2 and NMSS002, which showed signals of inbreeding (over 50 cM of their genome in ROH spanning >20 cM), and Cishan samples with low sequencing depth (<0.3×) were excluded from the analysis. (supplementary table S1a and S3, Supplementary Material online).

### Admixture Modeling with qpAdm and qpGraph

QpAdm (Patterson et al. 2006, 2012) analysis was applied to model the ancestries of ancient and modern pigs (supplementary tables S1a, S1c and S4, Supplementary Material online). We used Sumatran wild boar, Barbatus, Near East wild boar, Xuecheng, and Early North (with Miaodigou excluded) as the standard set of right populations. Miaodigou, Late North, Haimenkou, and the West commercial breeds were used as potential sources. The individuals included in each group are listed in supplementary tables S1a and S1c, Supplementary Material online. The source population combinations were tested by qpWave (Patterson et al. 2006, 2012) to ensure the right population set could distinguish all the source populations. For Southern China modern pigs (Luchuan, Bama, Wuzhishan), we replaced the Sumatran wild boar with *Sus verrucosus* from the right population and used the Sumatran wild boar as a source population (supplementary table S4c, Supplementary Material online).

QpGraph (Patterson et al. 2006, 2012) modeling was carried out using allsnps mode. We first constructed an initial topology comprising four populations (Sumatran, West Eurasia, Early North, and Late North). Subsequently, we used a custom Python script to incrementally add additional populations to the existing structure. The script generated all possible 1-way, 2-way, and 3-way admixture models, and the best-fitting topology was selected based on the worst *Z* scores. When multiple valid models were identified, we prioritized those with lower |*Z*| values and fewer admixture events as the locally optimal topology. A threshold of |*Z*| ≤ 4 instead of |*Z*| ≤ 3 was used to incorporate all populations in one graph due to the model complexity. Details of the step-wise qpGraph analysis are provided in supplementary material section S7, Supplementary Material online. As revealed by ADMIXTURE (Fig. 1d and supplementary fig. S4, Supplementary Material online) and qpAdm analysis (Fig. 3c, supplementary fig. S11, supplementary table S4, Supplementary Material online), some modern Chinese breeds may have experienced extensive influence from the commercial pig breeds in Western Eurasia. This may exaggerate gene flow from Western Eurasia, thereby affecting our reconstruction of the overall migration and admixture processes of Chinese domestic pigs. To address this, we excluded breeds with high Western Eurasian genetic components, including Min pig, Yunnan Tibetan, and Tibet Tibetan. Jicha was not included in the analysis, as it was a single individual with low sequencing depth (0.5×). At the same time, considering that Xuecheng exhibited a genetic background similar to that of northern domestic pigs (Early North) in the population clustering analysis, we included Xuecheng in the Early North group to increase the sample size for Early North in this analysis. Details of the populations used in this analysis are provided in supplementary tables S1a and S1c, Supplementary Material online.

### Coat Color Gene Analysis

The black coat phenotype in Chinese domestic pig resulted from an *MC1R* gene variant (Chr6:181,883 A > G) (Fang et al. 2009). We counted the reads carrying reference or alternative alleles using BCFtools v1.15.1, and estimated group-based allele frequencies at this site using a maximum likelihood approach described in a previous study (Mathieson et al. 2015) for ancient and modern samples (Fig. 4a, supplementary tables S5a, S5b and S5c, Supplementary Material online). The group information, read counts, and estimated allele frequencies are provided in supplementary tables S5a, S5b and S5c, Supplementary Material online, respectively.

For haplotype analysis, chromosome 6 was imputed and phased with GLIMPSE v1.1.1 (Rubinacci et al. 2021), with the SWIM reference dataset, and BCFtools was used for genotype likelihood (GL) calculation. Sequences of ancient samples were treated differently according to their library types to remove deamination. For partial-UDG double-stranded libraries, we trimmed 1 to 3 bp from both ends of reads according to the damage pattern profile using trimBam in bamutils (Jun et al. 2015 ). For non-UDG double-stranded libraries, we used untrimmed BAM files and set all transition sites as missing. For single-stranded libraries, we separated forward and reverse reads prior to GL calculation. During GL estimation, we included forward reads for G/A variants, reverse reads for C/T variants, and used all reads for transversion variants, integrating these results for imputation. After imputation, we calculated the genotype posterior probability (GP) for each sample and retained 19 individuals with an average GP > 0.9 for subsequent analyses (supplementary tables S5a, S5d and S5e, Supplementary Material online). Alternative allele frequencies of Chr6:181,883 A > G were calculated for each group, including samples with GP > 0.95. The haplotype network of the *MC1R* gene (Chr6:181,225 to 182,187) was constructed in DnaSP v6.12.02 (Fig. 4b, supplementary fig. S20, Supplementary Material online). We also plotted a haplotype heatmap across a 25 kb *MC1R*-flanking region (Chr6:156,225 to 207,187) (supplementary figs. S19 to S20, supplementary table S5e, Supplementary Material online). Details of the analysis are provided in supplementary material section S8, Supplementary Material online.

## Supplementary Material

msaf214_Supplementary_Data

## Data Availability

The aligned sequences of all newly reported samples are available at the Genome Sequence Archive (https://ngdc.cncb.ac.cn/gsa/) under the accession number PRJCA038667.

## References

[msaf214-B1] Alexander DH, Novembre J, Lange K. Fast model-based estimation of ancestry in unrelated individuals. Genome Res. 2009:19(9):1655–1664. 10.1101/gr.094052.109. 19648217 PMC2752134

[msaf214-B2] Andrews S . FastQC: a quality control tool for high throughput sequence data. 2010. http://www.bioinformatics.babraham.ac.uk/projects/fastqc/ (accessed on 17 Apr 2024).

[msaf214-B3] Boessenkool S, Hanghøj K, Nistelberger HM, Der Sarkissian C, Gondek AT, Orlando L, Barrett JH, Star B. Combining bleach and mild predigestion improves ancient DNA recovery from bones. Mol Ecol Resour. 2017:17(4):742–751. 10.1111/1755-0998.12623. 27790833

[msaf214-B4] Briggs AW, Stenzel U, Meyer M, Krause J, Kircher M, Pääbo S. Removal of deaminated cytosines and detection of in vivo methylation in ancient DNA. Nucleic Acids Res. 2010:38(6):e87–e87. 10.1093/nar/gkp1163. 20028723 PMC2847228

[msaf214-B5] Canu A, Vilaça ST, Iacolina L, Apollonio M, Bertorelle G, Scandura M. Lack of polymorphism at the MC1R wild-type allele and evidence of domestic allele introgression across European wild boar populations. Mamm Biol. 2016:81(5):477–479. 10.1016/j.mambio.2016.01.003.

[msaf214-B6] Capella-Gutiérrez S, Silla-Martínez JM, Gabaldón T. Trimal: a tool for automated alignment trimming in large-scale phylogenetic analyses. Bioinformatics. 2009:25(15):1972–1973. 10.1093/bioinformatics/btp348. 19505945 PMC2712344

[msaf214-B7] Carøe C, Gopalakrishnan S, Vinner L, Mak SS, Sinding MHS, Samaniego JA, Wales N, Sicheritz-Pontén T, Gilbert MTP. Single-tube library preparation for degraded DNA. Methods Ecol Evol. 2018:9(2):410–419. 10.1111/2041-210X.12871.

[msaf214-B8] Chen H, Huang M, Yang B, Wu Z, Deng Z, Hou Y, Ren J, Huang L. Introgression of eastern Chinese and southern Chinese haplotypes contributes to the improvement of fertility and immunity in European modern pigs. Gigascience. 2020:9(3):giaa014. 10.1093/gigascience/giaa014. 32141510 PMC7059266

[msaf214-B9] Cucchi T, Hulme-Beaman A, Yuan J, Dobney K. Early Neolithic pig domestication at Jiahu, Henan Province, China: clues from molar shape analyses using geometric morphometric approaches. J Archaeol Sci. 2011:38(1):11–22. 10.1016/j.jas.2010.07.024.

[msaf214-B10] Dabney J, Knapp M, Glocke I, Gansauge M-T, Weihmann A, Nickel B, Valdiosera C, García N, Pääbo S, Arsuaga J-L, et al Complete mitochondrial genome sequence of a Middle Pleistocene cave bear reconstructed from ultrashort DNA fragments. Proceedings of the National Academy of Sciences. 2013:110(39):15758–15763. 10.1073/pnas.1314445110. PMC3785785 24019490

[msaf214-B11] Daley T, Smith AD. Predicting the molecular complexity of sequencing libraries. Nat Methods. 2013:10(4):325–327. 10.1038/nmeth.2375. 23435259 PMC3612374

[msaf214-B12] Danecek P, Bonfield JK, Liddle J, Marshall J, Ohan V, Pollard MO, Whitwham A, Keane T, McCarthy SA, Davies RM, et al Twelve years of SAMtools and BCFtools. Gigascience. 2021:10(2):giab008. 10.1093/gigascience/giab008. 33590861 PMC7931819

[msaf214-B13] Ding R, Savegnago R, Liu J, Long N, Tan C, Cai G, Zhuang Z, Wu J, Yang M, Qiu Y, et al The SWine IMputation (SWIM) haplotype reference panel enables nucleotide resolution genetic mapping in pigs. Commun Biol. 2023:6(1):577. 10.1038/s42003-023-04933-9. 37253973 PMC10229620

[msaf214-B14] Dong N, Yuan J. Rethinking pig domestication in China: regional trajectories in central China and the Lower Yangtze Valley. Antiquity. 2020:94(376):864–879. 10.15184/aqy.2020.122.

[msaf214-B15] Driscoll CA, Menotti-Raymond M, Roca AL, Hupe K, Johnson WE, Geffen E, Harley EH, Delibes M, Pontier D, Kitchener AC, et al The near eastern origin of cat domestication. Science. 2007:317(5837):519–523. 10.1126/science.1139518. 17600185 PMC5612713

[msaf214-B16] Edgar RC . Muscle5: high-accuracy alignment ensembles enable unbiased assessments of sequence homology and phylogeny. Nat Comm. 2022:13(1):6968. 10.1038/s41467-022-34630-w. PMC9664440 36379955

[msaf214-B17] Ewels P, Magnusson M, Lundin S, Käller M. MultiQC: summarize analysis results for multiple tools and samples in a single report. Bioinformatics. 2016:32(19):3047–3048. 10.1093/bioinformatics/btw354. 27312411 PMC5039924

[msaf214-B18] Ewels PA, Peltzer A, Fillinger S, Patel H, Alneberg J, Wilm A, Garcia MU, Di Tommaso P, Nahnsen S. The nf-core framework for community-curated bioinformatics pipelines. Nat Biotechnol. 2020:38(3):276–278. 10.1038/s41587-020-0439-x. 32055031

[msaf214-B19] Excoffier L, Lischer HEL. Arlequin suite ver 3.5: a new series of programs to perform population genetics analyses under Linux and Windows. Mol Ecol Resour. 2010:10(3):564–567. 10.1111/j.1755-0998.2010.02847.x. 21565059

[msaf214-B20] Fang MY, Larson G, Ribeiro HS, Li Ning LN, Andersson L. Contrasting mode of evolution at a coat color locus in wild and domestic pigs. PLoS Genet. 2009:5(1):e1000341. 10.1371/journal.pgen.1000341. 19148282 PMC2613536

[msaf214-B21] Fontanesi L, Ribani A, Scotti E, Utzeri VJ, Veličković N, Dall’Olio S. Differentiation of meat from European wild boars and domestic pigs using polymorphisms in the MC1R and NR6A1 genes. Meat Sci. 2014:98(4):781–784. 10.1016/j.meatsci.2014.07.026. 25134014

[msaf214-B22] Frantz LA, Bradley DG, Larson G, Orlando L. Animal domestication in the era of ancient genomics. Nat Rev Genet. 2020:21(8):449–460. 10.1038/s41576-020-0225-0. 32265525

[msaf214-B23] Frantz LA, Haile J, Lin AT, Scheu A, Geörg C, Benecke N, Alexander M, Linderholm A, Mullin VE, Daly KG, et al Ancient pigs reveal a near-complete genomic turnover following their introduction to Europe. Proc Natl Acad Sci USA. 2019:116(35):17231–17238. 10.1073/pnas.1901169116. 31405970 PMC6717267

[msaf214-B24] Frantz LA, Schraiber JG, Madsen O, Megens H-J, Cagan A, Bosse M, Paudel Y, Crooijmans RP, Larson G, Groenen MA. Evidence of long-term gene flow and selection during domestication from analyses of Eurasian wild and domestic pig genomes. Nat Genet. 2015:47(10):1141–1148. 10.1038/ng.3394. 26323058

[msaf214-B25] Giuffra E, Kijas JMH, Amarger V, Carlborg Ö, Jeon J-T, Andersson L. The origin of the domestic pig: independent domestication and subsequent introgression. Genetics. 2000:154(4):1785–1791. 10.1093/genetics/154.4.1785. 10747069 PMC1461048

[msaf214-B26] Groenen MA, Archibald AL, Uenishi H, Tuggle CK, Takeuchi Y, Rothschild MF, Rogel-Gaillard C, Park C, Milan D, Megens H-J, et al Analyses of pig genomes provide insight into porcine demography and evolution. Nature. 2012:491(7424):393–398. 10.1038/nature11622. 23151582 PMC3566564

[msaf214-B27] Jónsson H, Ginolhac A, Schubert M, Johnson PL, Orlando L. mapDamage2. 0: fast approximate Bayesian estimates of ancient DNA damage parameters. Bioinformatics. 2013:29(13):1682–1684. 10.1093/bioinformatics/btt193. 23613487 PMC3694634

[msaf214-B28] Joseph TA, Pe’er I. Inference of population structure from time-series genotype data. Am J Hum Genet. 2019:105(2):317–333. 10.1016/j.ajhg.2019.06.002. 31256878 PMC6698887

[msaf214-B29] Jun G, Wing MK, Abecasis GR, Kang HM. An efficient and scalable analysis framework for variant extraction and refinement from population-scale DNA sequence data. Genome Res. 2015:25(6):918–925. 10.1101/gr.176552.114. 25883319 PMC4448687

[msaf214-B30] Kapp JD, Green RE, Shapiro B. A fast and efficient single-stranded genomic library preparation method optimized for ancient DNA. J Hered. 2021:112(3):241–249. 10.1093/jhered/esab012. 33768239 PMC8141684

[msaf214-B31] Korostin D, Kulemin N, Naumov V, Belova V, Kwon D, Gorbachev A. Comparative analysis of novel MGISEQ-2000 sequencing platform vs Illumina HiSeq 2500 for whole-genome sequencing. PLoS One. 2020:15(3):e0230301. 10.1371/journal.pone.0230301. 32176719 PMC7075590

[msaf214-B32] Larson G, Karlsson EK, Perri A, Webster MT, Ho SY, Peters J, Stahl PW, Piper PJ, Lingaas F, Fredholm M, et al Rethinking dog domestication by integrating genetics, archeology, and biogeography. Proc Natl Acad Sci USA. 2012:109(23):8878–8883. 10.1073/pnas.1203005109. 22615366 PMC3384140

[msaf214-B33] Larson G, Liu R, Zhao X, Yuan J, Fuller D, Barton L, Dobney K, Fan Q, Gu Z, Liu X-H, et al Patterns of east Asian pig domestication, migration, and turnover revealed by modern and ancient DNA. Proc Natl Acad Sci USA. 2010:107(17):7686–7691. 10.1073/pnas.0912264107. 20404179 PMC2867865

[msaf214-B34] Lazaridis I, Nadel D, Rollefson G, Merrett DC, Rohland N, Mallick S, Fernandes D, Novak M, Gamarra B, Sirak K, et al Genomic insights into the origin of farming in the ancient near east. Nature. 2016:536(7617):419–424. 10.1038/nature19310. 27459054 PMC5003663

[msaf214-B35] Leigh JW, Bryant D, Nakagawa S. POPART: full-feature software for haplotype network construction. Methods Ecol Evol. 2015:6(9):1110–1116. 10.1111/2041-210X.12410.

[msaf214-B36] Li H . 2013. Aligning sequence reads, clone sequences and assembly contigs with BWA-MEM [preprint], arXiv, arXiv:1303.3997.

[msaf214-B37] Li H, Durbin R. Fast and accurate short read alignment with burrows–wheeler transform. Bioinformatics. 2009:25(14):1754–1760. 10.1093/bioinformatics/btp324. 19451168 PMC2705234

[msaf214-B38] Li H, Handsaker B, Wysoker A, Fennell T, Ruan J, Homer N, Marth G, Abecasis G, Durbin R. Subgroup 1000 genome project data processing. 2009. The sequence alignment/map format and SAMtools. Bioinformatics. 2009:25(16):2078–2079. 10.1093/bioinformatics/btp352. 19505943 PMC2723002

[msaf214-B39] Li M, Tian S, Jin L, Zhou G, Li Y, Zhang Y, Wang T, Yeung CK, Chen L, Ma J, et al Genomic analyses identify distinct patterns of selection in domesticated pigs and Tibetan wild boars. Nat Genet. 2013:45(12):1431–1438. 10.1038/ng.2811. 24162736

[msaf214-B40] Librado P, Orlando L. Struct-f4: a Rcpp package for ancestry profile and population structure inference from f 4-statistics. Bioinformatics. 2022:38(7):2070–2071. 10.1093/bioinformatics/btac046. 35080599 PMC8963280

[msaf214-B41] Librado P, Tressières G, Chauvey L, Fages A, Khan N, Schiavinato S, Calvière-Tonasso L, Kusliy MA, Gaunitz C, Liu X, et al Widespread horse-based mobility arose around 2200 BCE in Eurasia. Nature. 2024:631(8022):819–825. 10.1038/s41586-024-07597-5. 38843826 PMC11269178

[msaf214-B42] Linderholm A, Larson G. The role of humans in facilitating and sustaining coat colour variation in domestic animals. Semin Cell Dev Biol. 2013:24(6-7):587–593. 10.1016/j.semcdb.2013.03.015. 23567209

[msaf214-B43] Linderholm A, Spencer D, Battista V, Frantz L, Barnett R, Fleischer RC, James HF, Duffy D, Sparks JP, Clements DR, et al A novel *MC1R* allele for black coat colour reveals the Polynesian ancestry and hybridization patterns of Hawaiian feral pigs. R Soc Open Sci. 2016:3(9):160304. 10.1098/rsos.160304. 27703696 PMC5043315

[msaf214-B44] Liu C-C, Witonsky D, Gosling A, Lee JH, Ringbauer H, Hagan R, Patel N, Stahl R, Novembre J, Aldenderfer M, et al Ancient genomes from the Himalayas illuminate the genetic history of Tibetans and their Tibeto-Burman speaking neighbors. Nat Commun. 2022:13(1):1203. 10.1038/s41467-022-28827-2. 35260549 PMC8904508

[msaf214-B45] Liu Y, Li T, Fan W, Hou Y, Song H. The subsistence strategy transformation of the Yangshao culture (6900-4800BP) in the Guanzhong area and western Henan based on new faunal materials from the Miaodigou site. J Archaeol Sci Rep. 2024:58:104725. 10.1016/j.jasrep.2024.104725.

[msaf214-B46] Loog L, Thomas MG, Barnett R, Allen R, Sykes N, Paxinos PD, Lebrasseur O, Dobney K, Peters J, Manica A, et al Inferring allele frequency trajectories from ancient DNA indicates that selection on a chicken gene coincided with changes in medieval husbandry practices. Mol Biol Evol. 2017:34(8):1981–1990. 10.1093/molbev/msx142. 28444234 PMC5850110

[msaf214-B47] Mathieson I, Lazaridis I, Rohland N, Mallick S, Patterson N, Roodenberg SA, Harney E, Stewardson K, Fernandes D, Novak M, et al Genome-wide patterns of selection in 230 ancient Eurasians. Nature. 2015:528(7583):499–503. 10.1038/nature16152. 26595274 PMC4918750

[msaf214-B48] Modi A, Vai S, Caramelli D, Lari M. The Illumina sequencing protocol and the NovaSeq 6000 system. In: Mengoni A, Bacci G, Fondi M, editors. Bacterial pangenomics: methods and protocols. New York, NY: Humana Press, an imprint of Springer Nature; 2021. p. 15–42. 10.1007/978-1-0716-1099-2. 33961215

[msaf214-B49] Neukamm J, Peltzer A, Nieselt K. DamageProfiler: fast damage pattern calculation for ancient DNA. Bioinformatics. 2021:37(20):3652–3653. 10.1093/bioinformatics/btab190. 33890614

[msaf214-B50] Nguyen L-T, Schmidt HA, Von Haeseler A, Minh BQ. IQ-TREE: a fast and effective stochastic algorithm for estimating maximum-likelihood phylogenies. Mol Biol Evol. 2015:32(1):268–274. 10.1093/molbev/msu300. 25371430 PMC4271533

[msaf214-B51] Okonechnikov K, Conesa A, García-Alcalde F. Qualimap 2: advanced multi-sample quality control for high-throughput sequencing data. Bioinformatics. 2016:32(2):292–294. 10.1093/bioinformatics/btv566. 26428292 PMC4708105

[msaf214-B52] Olden JD, Poff NL, Douglas MR, Douglas ME, Fausch KD. Ecological and evolutionary consequences of biotic homogenization. Trends Ecol Evol. 2004:19(1):18–24. 10.1016/j.tree.2003.09.010. 16701221

[msaf214-B53] Ottoni C, Girdland Flink L, Evin A, Geörg C, De Cupere B, Van Neer W, Bartosiewicz L, Linderholm A, Barnett R, Peters J, et al Pig domestication and human-mediated dispersal in western Eurasia revealed through ancient DNA and geometric morphometrics. Mol Biol Evol. 2013:30(4):824–832. 10.1093/molbev/mss261. 23180578 PMC3603306

[msaf214-B54] Patterson N, Moorjani P, Luo Y, Mallick S, Rohland N, Zhan Y, Genschoreck T, Webster T, Reich D. Ancient admixture in human history. Genetics. 2012:192(3):1065–1093. 10.1534/genetics.112.145037. 22960212 PMC3522152

[msaf214-B55] Patterson N, Price AL, Reich D. Population structure and eigenanalysis. PLoS Genet. 2006:2(12):e190. 10.1371/journal.pgen.0020190. 17194218 PMC1713260

[msaf214-B56] Peng Y, Cai X, Wang Y, Liu Z, Zhao Y. Genome-wide analysis suggests multiple domestication events of Chinese local pigs. Anim Genet. 2022:53(3):293–306. 10.1111/age.13183. 35277870

[msaf214-B57] Price AL, Patterson NJ, Plenge RM, Weinblatt ME, Shadick NA, Reich D. Principal components analysis corrects for stratification in genome-wide association studies. Nat Genet. 2006:38(8):904–909. 10.1038/ng1847. 16862161

[msaf214-B58] Purcell S, Neale B, Todd-Brown K, Thomas L, Ferreira MA, Bender D, Maller J, Sklar P, De Bakker PI, Daly MJ, et al PLINK: a tool set for whole-genome association and population-based linkage analyses. Am J Hum Genet. 2007:81(3):559–575. 10.1086/519795. 17701901 PMC1950838

[msaf214-B59] Reimer PJ, Austin WE, Bard E, Bayliss A, Blackwell PG, Ramsey CB, Butzin M, Cheng H, Edwards RL, Friedrich M, et al The IntCal20 northern Hemisphere radiocarbon age calibration curve (0–55 cal kBP). Radiocarbon. 2020:62(4):725–757. 10.1017/RDC.2020.41.

[msaf214-B60] Renaud G, Slon V, Duggan AT, Kelso J. Schmutzi: estimation of contamination and endogenous mitochondrial consensus calling for ancient DNA. Genome Biol. 2015:16(1):224. 10.1186/s13059-015-0776-0. 26458810 PMC4601135

[msaf214-B61] Retief JD . Phylogenetic analysis using PHYLIP. In: Misener S, Krawetz SA, editors. Bioinformatics methods and protocols. 132. New Jersey, US: Humana Press; 1999. p. 243–258. 10.1385/1-59259-192-2:243. 10547839

[msaf214-B62] Ringbauer H, Novembre J, Steinrücken M. Parental relatedness through time revealed by runs of homozygosity in ancient DNA. Nat Commun. 2021:12(1):5425. 10.1038/s41467-021-25289-w. 34521843 PMC8440622

[msaf214-B63] Rohland N, Harney E, Mallick S, Nordenfelt S, Reich D. Partial uracil–DNA–glycosylase treatment for screening of ancient DNA. Philos Trans R Soc Lond B Biol Sci. 2015:370(1660):20130624. 10.1098/rstb.2013.0624. 25487342 PMC4275898

[msaf214-B64] Rossi C, Sinding M-HS, Mullin VE, Scheu A, Erven JA, Verdugo MP, Daly KG, Ciucani MM, Mattiangeli V, Teasdale MD, et al The genomic natural history of the aurochs. Nature. 2024:635(8037):136–141. 10.1038/s41586-024-08112-6. 39478219

[msaf214-B65] Rozas J, Ferrer-Mata A, Sánchez-DelBarrio JC, Guirao-Rico S, Librado P, Ramos-Onsins SE, Sánchez-Gracia A. DnaSP 6: DNA sequence polymorphism analysis of large data sets. Mol Biol Evol. 2017:34(12):3299–3302. 10.1093/molbev/msx248. 29029172

[msaf214-B66] Rubinacci S, Ribeiro DM, Hofmeister RJ, Delaneau O. Efficient phasing and imputation of low-coverage sequencing data using large reference panels. Nat Genet. 2021:53(1):120–126. 10.1038/s41588-020-00756-0. 33414550

[msaf214-B67] Schubert M, Lindgreen S, Orlando L. AdapterRemoval v2: rapid adapter trimming, identification, and read merging. BMC Res Notes. 2016:9(1):1–7. 10.1186/s13104-016-1900-2. 26868221 PMC4751634

[msaf214-B68] Tao L, Yuan H, Zhu K, Liu X, Guo J, Min R, He H, Cao D, Yang X, Zhou Z, et al Ancient genomes reveal millet farming-related demic diffusion from the Yellow River into southwest China. Curr Biol. 2023:33(22):4995–5002.e7. 10.1016/j.cub.2023.09.055. 37852263

[msaf214-B69] Wang C-C, Yeh H-Y, Popov AN, Zhang H-Q, Matsumura H, Sirak K, Cheronet O, Kovalev A, Rohland N, Kim AM, et al Genomic insights into the formation of human populations in east Asia. Nature. 2021a:591(7850):413–419. 10.1038/s41586-021-03336-2. 33618348 PMC7993749

[msaf214-B70] Wang H, Yang MA, Wangdue S, Lu H, Chen H, Li L, Dong G, Tsring T, Yuan H, He W, et al Human genetic history on the Tibetan Plateau in the past 5100 years. Sci Adv. 2023:9(11):eadd5582. 10.1126/sciadv.add5582. 36930720 PMC10022901

[msaf214-B71] Wang J, Tang Y, Zheng Y, Jiang L, Ma X, Hou Y, Sun G. Early evidence for pig domestication (8,000 cal. BP) in the lower Yangtze, south China. Proc Natl Acad Sci U S A. 2025:122(24):e2507123122. 10.1073/pnas.2507123122. 40489608 PMC12184640

[msaf214-B72] Wang T, Wang W, Xie G, Li Z, Fan X, Yang Q, Wu X, Cao P, Liu Y, Yang R, et al Human population history at the crossroads of east and Southeast Asia since 11,000 years ago. Cell. 2021b:184(14):3829–3841.e21. 10.1016/j.cell.2021.05.018. 34171307

[msaf214-B73] Wen J, Zheng Z, Gong M, Li D, Hu S, Cai Y, Wang Y, Nanaei HA, Zhang N, Yu T, et al Ancient genomes reveal the genetic inheritance and recent introgression in Chinese indigenous pigs. Sci China Life Sci. 2022:65(4):842–845. 10.1007/s11427-021-2066-y. 35201529

[msaf214-B74] White S . From globalized pig breeds to capitalist pigs: a study in animal cultures and evolutionary history. Environ Hist Durh N C. 2011:16(1):94–120. 10.1093/envhis/emq143.

[msaf214-B75] Wu G-S, Yao Y-G, Qu K-X, Ding Z-L, Li H, Palanichamy MG, Duan Z-Y, Li N, Chen Y-S, Zhang Y-P. Population phylogenomic analysis of mitochondrial DNA in wild boars and domestic pigs revealed multiple domestication events in east Asia. Genome Biol. 2007:8(11):1–12. 10.1186/gb-2007-8-11-r245. PMC2258183 18021448

[msaf214-B76] Yang B, Cui L, Perez-Enciso M, Traspov A, Crooijmans RPMA, Zinovieva N, Schook LB, Archibald A, Gatphayak K, Knorr C, et al Genome-wide SNP data unveils the globalization of domesticated pigs. Genet Sel Evol. 2017:49(1):71. 10.1186/s12711-017-0345-y. 28934946 PMC5609043

[msaf214-B77] Yang J, Zhang D, Yang X, Wang W, Perry L, Fuller DQ, Li H, Wang J, Ren L, Xia H, et al Sustainable intensification of millet–pig agriculture in Neolithic north China. Nat Sustain. 2022:5(9):780–786. 10.1038/s41893-022-00905-9.

[msaf214-B78] Yang MA, Fan X, Sun B, Chen C, Lang J, Ko Y-C, Tsang C, Chiu H, Wang T, Bao Q, et al Ancient DNA indicates human population shifts and admixture in northern and southern China. Science. 2020:369(6501):282–288. 10.1126/science.aba0909. 32409524

[msaf214-B79] Yates JAF, Lamnidis TC, Borry M, Valtueña AA, Fagernäs Z, Clayton S, Garcia MU, Neukamm J, Peltzer A. Reproducible, portable, and efficient ancient genome reconstruction with nf-core/eager. PeerJ. 2021:9:e10947. 10.7717/peerj.10947. 33777521 PMC7977378

[msaf214-B80] You Y, Chen X, Hein A, Qin C, Zhao Y, Zhang J, Liu T, Fan W, Yuan G. Pig domestication and human subsistence at the early Neolithic site of Guanjia (6100–5500 BC), central China. Archaeol Anthropol Sci. 2024:16(3):40. 10.1007/s12520-024-01941-6.

[msaf214-B81] Yuan J, Flad RK. Pig domestication in ancient China. Antiquity. 2002:76(293):724–732. 10.1017/S0003598X00091171.

[msaf214-B82] Yuan J, Jian-Lin H, Blench R. Livestock in ancient China: an archaeozoological perspective. In: Sanchez-Mazas A, Blench R, Ross MD, Peiros I, Lin M, editors. Past human migrations in east Asia: matching archaeology, linguistics and genetics. 1. Abingdon, Oxon, and New York, NY: Routledge; 2008. p. 84–104. 10.4324/9780203926789-17.

[msaf214-B83] Zeder MA . The domestication of animals. J Anthropol Res. 2012:68(2):161–190. 10.3998/jar.0521004.0068.201.

[msaf214-B84] Zhang M, Liu Y, Li Z, Lü P, Gardner JD, Ye M, Wang J, Yang M, Shao J, Wang W, et al Ancient DNA reveals the maternal genetic history of east Asian domestic pigs. J Genet Genomics. 2022:49(6):537–546. 10.1016/j.jgg.2021.11.014. 34902603

[msaf214-B85] Zhang Q, Hou Y, Li X, Styring A, Lee-Thorp J. Stable isotopes reveal intensive pig husbandry practices in the middle Yellow River region by the Yangshao period (7000–5000 BP). PLoS One. 2021:16(10):e0257524. 10.1371/journal.pone.0257524. 34610013 PMC8491901

[msaf214-B86] Zhang W, Yang M, Wang Y, Wu X, Zhang X, Ding Y, Yin Z. Genomic analysis reveals selection signatures of the Wannan Black pig during domestication and breeding. Asian-Australas J Anim Sci. 2020:33(5):712. 10.5713/ajas.19.0289. 31480149 PMC7206397

