# Supplementary material for: Ancient Genomics Reveals the Origin, Dispersal, and Human Management of East Asian Domestic Pigs
Source: Mol Biol Evol. 2025 Sep 25;42(9):msaf214. doi: 10.1093/molbev/msaf214 (PMC12461208; doi:10.1093/molbev/msaf214)
Supplement: msaf214_Supplementary_Data [file msaf214_supplementary_data.zip › Pig_MBE_supp_final.pdf]

Supplementary Material for

Ancient genomics reveals the origin, dispersal and human management of East Asian domestic pigs

Yu Han[1,2,†], Zhihan Zhao[1,3,†], David W.G. Stanton[4], Xiaole Lei[1,5,6], Zhuang Wu[7], Yiting Liu[8], Chong Yu[9], Xi Chen[10], Wenyan Li[11], Juan Wang[12], Yue You[13], Yue Li[14], Sha Lei[15], Hailin Yi[16], Wenquan Fan[17], Quanfa Cai[17], Rui Min[18], Changcheng Hu[18,19], Canping Chen[7], Yingjie Cui[7,20], Jiqiao Guo[21], Hongliang Zhang[22,23], Haichao Song[8], Xin Guo[24], Qiurong Ruan[25], Yuhua Tan[26], Ziyi Li[27], Xiangyu Zhang[28], Xingyu Shi[1], Xu Zhou[1], Yan Zhuang[1,28], Aurelie Manin[2,29], Laurent A. F. Frantz[2,30], Joel M. Alves[2,31,32], Yan Pan[33], Xiaohong Wu[33], Shu-Jin Luo[1,28], Greger Larson[2]*, He Yu[1]*

[*] Corresponding author. Email: yuhe@pku.edu.cn (H.Y.), greger.larson@arch.ox.ac.uk (G.L.)

The PDF includes:

Supplementary Text Section 1 to Section 9

Supplementary figs. S1 to S20

Table S3

Supplementary References

Legends for supplementary tables S1, S2, S4 and S5

Table of Contents

Section 1: Sample and archaeological site descriptions 3
Section 2: Mitochondrial phylogenetic analysis 10
Section 3: Autosomal phylogenetic analysis 13
Section 4: Unsupervised population clustering analyses 15
Section 5: Outgroup f_3 and f_4-statistics by archaeological sites 18
Section 6: Run of Homozygosity 21
Section 7: Admixture modeling using qpAdm and qpGraph 24
Section 8: The evolution of black coloration in Chinese domestic pigs 30
Section 9: A description of the dispersal map, related to (Fig. 5) 34
Supplementary References 36
Legends of supplementary tables S1, S2, S4 and S5 39

Section 1: Sample and archaeological site descriptions

New ancient samples and archaeological sites

Cishan

The Cishan site is located at the foothills of Drum Mountain in the Taihang Mountains, bordered to the north by the Cishan iron mine and to the south by the Ming River. Specifically, it is situated approximately 1 kilometer southeast of Cishan Village in Wu'an County, Hebei Province, standing 25 meters above the modern riverbed on a plateau approximately 20 kilometers southwest of the county. Radiocarbon dating showed that the site ranged from 8,100 to 7,800 years ago, belonging to the Middle Neolithic Age in China. Large accumulations of millets were discovered at the site, suggesting the rise of agriculture there (Rodwell 1984). *Sus* samples from the site are thought to be the earliest domestic pigs in Northern China (Yuan and Flad 2002). Four samples excavated from the site were analyzed in this study. Two of them (SS24 and SS26) yielded both nuclear and mitochondrial genomes, while the other two (SS17 and SS23) provided only mitochondrial genomes. Direct radiocarbon dating conducted on SS17 and SS26 revealed that they can be traced to 5,722 - 5,630 cal BCE and 5,878 - 5,721cal BCE respectively (Figs. 1A and 1B, supplementary tables S1A and S1B). Zooarchaeologists identified these four samples as domestic pigs according to their morphology. Accordingly, we classified the four samples as domestic pigs in our analysis.

Xuecheng

The Xuecheng site is located in Xuesi Village, Xuecheng Town, under the jurisdiction of Gaochun County, Nanjing City, Jiangsu Province, Chuanxi Town. The excavation revealed an area containing Neolithic tombs, ash pits, residential layouts, and hearths. Artifacts, including pottery, jade, stone tools, and animal remains were unearthed during the excavation (Zhou et al. 2000). One sample from this site, NJSS011, was used in this study. It was found in sacrificial context (Pit H31). The results of radiocarbon dating on a charcoal (H31-1) stratigraphically associated with the individual spans from 3,265 to 2,929 cal BCE (Figs. 1A and 1B, supplementary tables S1A and S1B). Based on the skeletal morphology and its unique archaeological context for sacrifice, zooarchaeologists have identified it as domestic pigs. Accordingly, we classify this specimen as domestic pig in our analysis as well.

Miaodigou

The Miaodigou site is located on the Loess Plateau between the Qinglongjian River and Canglongjian River, which both flow into the Yellow River, covering an area of approximately 240,000 square meters. The early phase of the site belongs to the Miaodigou Culture of the Yangshao Period, dating from around 6,000 to 5,000 years ago. The upper phase of the site is associated with the Longshan Culture of the Central Plain, dating to 4,900-4,800 years ago. The major subsistence at this site were agriculture and animal husbandry (Han 2013; Zhong et al. 2020; Liu et al. 2024). Two samples (SS2 and SS10) excavated on the site were used in this research. The two samples were directly radiocarbon dated to 2,935 - 2,875 cal BCE and 3,359 - 3262 cal BCE, respectively (Figs. 1A and 1B, supplementary tables S1A and S1B). Based on the age-at-death profile and skeletal morphology, researchers have determined that the *Sus scrofa* unearthed at this site primarily represent domestic pigs. Therefore, we treated the two samples obtained from this site as domestic pigs in our analysis.

Zheng and Han City

The Zheng-Han City was one of the capital cities during the Eastern Zhou Dynasty. The city was initially established by the State of Zheng during the Spring and Autumn Period (770 - 476 BCE). The State of Han conquered Zheng, and moved its capital to this location, with both Zheng and Han successively building their capitals here for a total of 539 years. The layout of the Zheng-Han Ancient City is an irregular triangle, with a north-south dividing wall in the middle that separates the city into eastern and western sections (Ma 1978). A domestic pig (SS13) and a wild boar (SS14) from the site were used in this study. The two samples were directly radiocarbon dated to 376 - 194 cal BCE and 670 - 451 cal BCE, respectively (Figs. 1A and 1B, supplementary tables S1A and S1B). According to the information provided by the zooarchaeologist, *Sus scrofa* from this site predominantly consists of domestic pigs, although there is still a notable proportion of wild boars. Based on the skeletal size of the samples, SS13 is considered likely to belong to a domestic pig, while SS14 is considered likely to belong to a wild boar.

Gongbeiya

The Gongbeiya site is located on the west bank of the Zao River, in the southwestern suburbs of Xi'an, Shaanxi Province. The excavation carried out in 2017 yielded 102

ash pits, 6 tombs, and 4 pottery kilns in an area of 800 square meters. Based on the analysis of the architecture and artifacts, the occupation of the Gongbeiya site was divided into four phases: the first phase corresponds to the Middle Yangshao period; the second phase to the Late Yangshao period; the third phase to the Eastern Zhou period; and the fourth phase to the Qin-Han period (Zong et al. 2021). One sample (XBSS002) was used in this research. The sample was originally believed, based on archaeological context, to be from the Qin-Han period (475 BCE - 220 CE). However, direct radiocarbon dating has since placed it between 771 and 522 cal BCE, indicating it actually dates to the earlier Zhou period (Figs. 1A and 1B, supplementary tables S1A and S1B). Based on the information provided by the zooarchaeologists, this sample is considered to belong to a domestic pig.

Shiyicheng

The Shiyicheng site is located in the northwest of Erguyi Village, Tongye Town, Luquan District, Shijiazhuang City, Hebei Province. Shiyicheng is divided into an eastern and a western section, with the larger eastern city serving as an industrial zone and the smaller western city functioning as a military camp, horse grounds, and governmental facilities. The excavation took place in 2017 within the eastern city, yielding artifacts related to copper industry, bone processing, and pottery producing (Li et al. 2020). Based on the analysis of the unearthed artifacts, the site was used from the Zhou Dynasty to the Western Han Dynasty. One sample (HBSS006) from the site was used in this study. It has been directly dated to 721 - 394 cal BCE (Figs. 1A and 1B, supplementary tables S1A and S1B). Based on the information provided by the zooarchaeologists, this sample is considered to belong to a domestic pig.

Zhucang

The Zhucang site is a tomb complex, located west of Zhucang Village in Ping Le Town, Mengjin District, Luoyang City, Henan Province. The main tomb, M722, is potentially the burial site of Emperor Shun of the Eastern Han Dynasty. Initial excavations at the site were conducted in 2009, covering an area of 4,700 square meters, with further active excavations from 2014 to 2017 expanding to nearly 8,000 square meters. Current research preliminarily dates the Zhucang M722 tomb complex to the middle to late Eastern Han period (approximately 100 to 220 CE). During the second field archaeology campaign, the animal remains unearthed from ash pits,

drainage ditches, and cellars were meticulously collected by hand. Based on the construction and usage period of the tomb complex, as well as the archaeological context of these animal remains, it is believed that they represent the discarded remains of daily food consumption by the residents surrounding the tomb complex (Yan et al. 2011; Wu and Zhang 2023). One sample (HNSS002) coming from these animal remains was included in this study. It has been directly radiocarbon dated to 245 - 402 cal CE (Figs. 1A and 1B, supplementary tables S1A and S1B). Based on the information provided by the zooarchaeologists, this sample is considered to belong to a domestic pig.

Xitucheng

The Xitucheng City site is located in the westernmost Xitucheng Village of Erhao Bu Township, Kangbao County, Zhangjiakou City, Hebei Province. The site lies at the convergence of five administrative regions: Kangbao County, Zhangbei County, Shangyi County in Hebei Province, and Shangdu County and Huade County in the Inner Mongolia Autonomous Region. The historical period of this urban site is attributed to the middle of the Jin Dynasty (1,115 - 1,234 CE) (Chen et al. 2014; Wu et al. 2020). Two samples (HBSS010 and HBSS013) were used in this study, among which HBSS010 has been directly radiocarbon dated to 1,150 - 1262 cal CE (Figs. 1A and 1B, supplementary tables S1A and S1B). Based on the information provided by the zooarchaeologist, these samples are considered to belong to domestic pigs.

Halehaxite

The Bronze Age site of Halehaxite is situated in the northwest part of China, near the contemporary village of Halehaxite in Xinyuan County, Ili Kazak Autonomous Prefecture, Xinjiang Uygur Autonomous Region. Located on the north bank of the Qiapu River, a tributary river in the upper Ili Valley within the eastern Tianshan Mountains, the site was excavated in 2020. Recognized as one of the earliest and best-preserved Bronze Age settlements in the East Tianshan area, it dates back to approximately 3,400 - 3,000 years ago. The site provides significant data for exploring subsistence strategies and the evolution of crop farming and animal husbandry. Initial analyses of the material culture suggest that Halehaxite exhibits strong cultural links with other sites within the Andronovo cultural network across the Eastern Eurasian Steppe. Notably, it shares connections with the Adunqiaolu and

Husta sites in the upper Boertala River Valley, and the Jirentaigoukou site in the upper reaches of the Ili River Valley (Yu et al. 2022). One sample (XJSS003) from the site was used in this study (Figs. 1A and 1B, supplementary tables S1A and S1B). Zooarchaelogists classified the sample to be a wild boar, considering the skeletal morphology as well as the lack of documented cases of pig domestication in the ancient period in Xinjiang.

Dalete

The Dalete site, located north of modern Pochengzi village in Bole, was a pivotal urban center on the northern Silk Road from the 10th to 14th centuries AD. Significant for its strategic positioning, Dalete played a crucial role in the trade networks that facilitated cultural and economic exchanges across Eurasia. Excavations conducted in 2017 revealed extensive archaeological layers, underscoring its historical importance (Li et al. 2022). Two wild boars (XJSS006, XJSS007) were used in this study. XJSS006 has been directly radiocarbon dated to 1,120 - 1,228 cal CE (Figs. 1A and 1B, supplementary tables S1A and S1B). Zooarchaelogists classified these samples to be wild boars, considering the skeletal morphology as well as the lack of documented cases of pig domestications in ancient periods in Xinjiang.

Jicha

The Jicha site is strategically located at the convergence of the Mekong, Yongchun, and Jicha rivers, serving as a direct and logical route of transmission. Excavations conducted in 2022 unveiled a cultural sequence spanning the Late Neolithic, Early and Late Bronze Ages, and the Iron Age. The Late Neolithic layer features pottery similar to that found both upstream at Karuo and downstream at Haimenkou. The Early Bronze Age is marked by distinctive painted pottery. The site saw reoccupation around 700 BCE during the Late Bronze Age, with the northern settlement area containing semi-subterranean structures likely used as animal pens. The last period of occupation occurred during the Early Iron Age (300–1 BCE), after which the site was abandoned, likely due to landslides triggered by heavy flood(Fu et al. 2024). One sample (YNSS012) from the site was used in this study. The sample has been directly radiocarbon dated to 1,732 - 1,521 cal BCE (Figs. 1A and 1B, supplementary tables S1A and S1B). Based on the information provided by the zooarchaeologist, this sample is considered to be from a wild boar.

Haimenkou

Haimenkou, located in Jianchuan County, Northwest Yunnan, is the largest prehistoric site in the region. It sits at an elevation of 2190 meters in the Jinsha River Basin, near the Heihui River flowing into Jianhu Lake. Since its discovery in 1957, Haimenkou has seen several excavations. Notably, the site has yielded both bronze and iron objects, pointing to early metal use and production in Yunnan. The chronological framework established by radiocarbon dating spans from the Neolithic to the Bronze Age, approximately 1,600 to 300 cal BCE, with significant cultural transitions observed through these periods.The faunal remains suggest a mix of domesticated and wild animals, including pigs, sheep/goats, dogs, and potentially gaur, hinting at complex subsistence strategies. The findings from Haimenkou, with its distinct cultural layers and artifacts, provide valuable insights into the prehistoric occupation and technological advancements in the region (Xue et al. 2022). Two samples (YNSS002 and YNSS007) stemming from the Neolithic or Bronze Age occupation phases of the site were used in this study. These two samples have been directly radiocarbon dated to 1,532 - 1,425 cal BCE and 650 - 543 cal BCE respectively (Figs. 1A and 1B, supplementary tables S1A and S1B). Based on the information provided by zooarchaeologists, these samples are considered domestic pigs.

Shikouzi

The Shikouzi site is located approximately 1000 meters southeast of Xiaotan Village, Dalu New District, in the Zhungeer Banner of the Inner Mongolia Autonomous Region. Positioned on the second-level terrace of the western bank of the Yellow River, the site sits atop a sandy ridge. Discovered in the 1960s, Shikouzi has been identified as a prehistoric site spanning from the late to the final phases of the Neolithic period. The primary cultural layer belongs to the Zhujiagou culture (~2,000 to 1,500 BCE). The site covers an excavation area of 800 square meters. The excavation has uncovered 39 ash pits from the Zhujiagou culture, one residential facility, and five tombs from the Eastern Zhou period. A diverse array of artifacts made from pottery, stone, copper, iron, and bone have been recovered (Xu et al. 2019). One sample (NMSS002) from the site was used in this study. It was directly radiocarbon dated to 1,421 - 1,262 cal BCE (Figs. 1A and 1B, supplementary tables

S1A and S1B). Based on the information provided by the zooarchaeologist, this sample is considered a domestic pig.

Dashuigou

The Dashuigou site, also known as Dengcaogou Liang site, is located on the terraces surrounding Dashuigou Village, Gaojiaying Town, Chongli County, Zhangjiakou City, Hebei Province. To its west lies the Qingshui River, and it is flanked by gullies on both the north and south sides. The site is approximately 2 kilometers southwest of the downtown area of Zhangjiakou. It was discovered during the third national survey of cultural relics. Investigation and trial excavation revealed that the site spans 151,500 square meters and primarily dates back to the Longshan culture period. During the trial excavations, four exploratory trenches were laid out, uncovering six ash-faced residential sites, four ash pits, and one ash trench. The cultural content and artifacts collected from the Dashuigou site bear similarities to those found in the Lao Hushan culture of the Daihai area and the Youyao culture of Xinzhou, Shanxi Province. These findings highlight its significance in studying the regional distribution and cultural exchanges during the Longshan period (Wu et al. 2017). Two samples from the site (HBSS003 and HBSS004) were used in this research. HBSS003 has been directly radiocarbon dated to 2,136 - 1,935 cal BCE (Figs. 1A and 1B, supplementary tables S1A and S1B). Based on the information provided by zooarchaeologists, these samples are considered a domestic pig.

Published resources

Published whole genome resequencing data of pigs and wild boars were downloaded from the NCBI Sequence Read Archive (SRA) database. This study has gathered data from 120 modern individuals and 2 ancient Chinese individuals.

The modern data originate from 18 projects: PRJNA255085, PRJNA369600, PRJEB9922 (Frantz et al. 2015), PRJNA550237 (Chen et al. 2020), PRJNA378496, PRJNA305081, SRP320424, PRJNA309108, PRJNA238851, PRJNA524263 (Zhang et al. 2020), PRJNA398176, SRP028348, PRJNA488327, PRJNA213179, PRJNA186497, PRJEB35180, and PRJEB1683. These include 26 native pigs from Northern China (Min n=6, Laiwu Black n=5, Hetao n= 5, Nanyang n=5, Bamei n=6); 26 native pigs from the Yangtze River Basin (Tongcheng n=4, Wanan Black n=6,

Meishan n=6, Jinhua n=3, Jiangquhai n=3, Erhualian n=4); 26 native pigs from Southwestern China (Tibetan pigs from Yunnan n=6, Tibetan pigs from Tibet n=6, Tibetan pigs from Sichuan n=6, Tibetan pigs from Gansu n=6, Rongchang n=2); 17 native pigs from Southern China (Wuzhishan n=6, Luchuan n=6, Bama n=5); 5 native wild boar from the Qinghai-Tibet Plateau; 13 West Eurasian wild boars and domestic pigs (Iberian pigs n=2, Near Eastern wild boars n=2, Italian wild boars n=3, Large White n=4, 2 Duroc n=4); 7 outgroup individuals (Sumatran wild boars n=2, Bornean bearded pigs *Sus barbatus* n=2, Javan warty pig *Sus verrucosus* n=1, and African warthog *Phacochoerus africanus* n=1). Details of the samples are shown in supplementary table S1C.

The genomic data from two ancient Chinese pigs from the Shimao site in Shaanxi, dated approximately 3,900 years ago was downloaded from project PRJNA788987 (Wen et al. 2022). Ancient mitochondrial genome sequences for 42 ancient East Asian pigs were collected from the GenBank database (Accession numbers: OL547441–OL547482) (supplementary table S1C) (Zhang et al. 2022).

Section 2: Mitochondrial phylogenetic analysis

In the maximum likelihood phylogenetic tree constructed using mitochondrial genomes (ML tree), Eastern and Western Eurasian samples diverged into two distinct clades with all ancient Chinese domestic pigs falling into the Eastern Eurasia clade (supplementary fig. S1), supporting an independent origin of Chinese domestic pigs and long-term matriline continuity as revealed by previous studies (Larson et al. 2010; Wen et al. 2022; Zhang et al. 2022). Some European Large White pigs were also located within the Eastern Eurasian clade, further demonstrating maternal genetic introgression from East Asian pigs into European commercial breeds (Yang et al. 2017; Frantz et al. 2019). The ancient wild boars from Xinjiang (Halehaxite and Dalete) (Figs. 1A and 1B, supplementary table S1A), although showing closer affinity to Western Eurasia in the nuclear DNA analysis (Fig. 1C), fell into the cluster of Eastern Eurasia. It indicated the unique position of Xinjiang population as the admixture of wild boars from the Eastern and Western Eurasia.

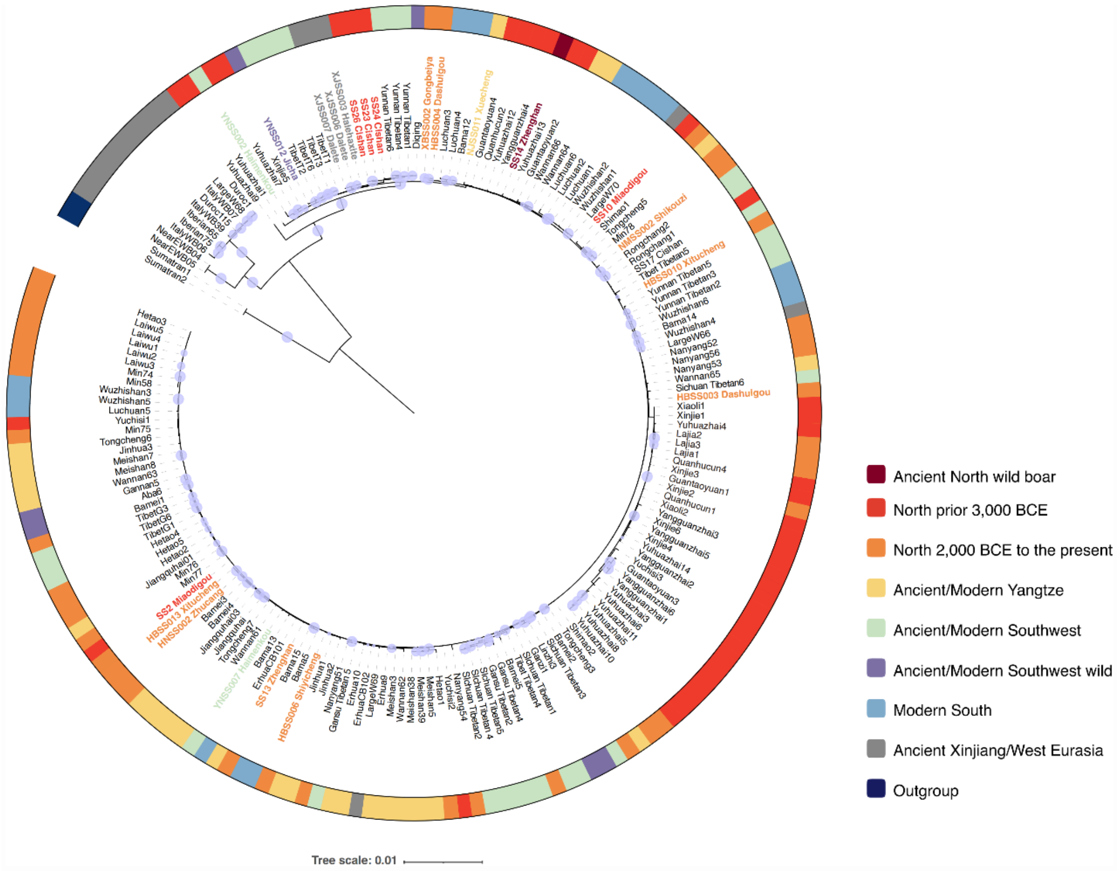

Supplementary fig. S1. Maximum likelihood Phylogenetic tree constructed using mitochondrial genomes. Size of the purple dots on branches indicate bootstrap values (>50-100). Tip labels of new ancient samples have been highlighted with different colors according to their groups. The newly reported ancient individuals have been highlighted with colors of the sample names corresponding to the color of dots in Fig. 1.

Pigs in Eastern Eurasia exhibited low spatial-temporal differentiations. The ancient domestic pigs/wild boars from various sites were distributed across different subclades without clear site-specific clustering (supplementary fig. S1). Modern individuals of the same regions and even the same breeds, did not group within the same cluster, and the statistical support for the branches within the Eastern Eurasia clade were usually low (bootstrap < 50) (supplementary fig. S1). We, therefore, think that mitochondrial genome data are insufficient to reveal the genetic structure of East Asian domestic pigs.

The haplotype network (supplementary fig. S2) supported the findings of the ML tree (supplementary fig. S1). It has further revealed that the ancient and modern domestic

pigs in East Asia have formed a major haplotype, without a clear spatiotemporal structure (supplementary fig. S2, supplementary tables S1A, S1B and S1D).

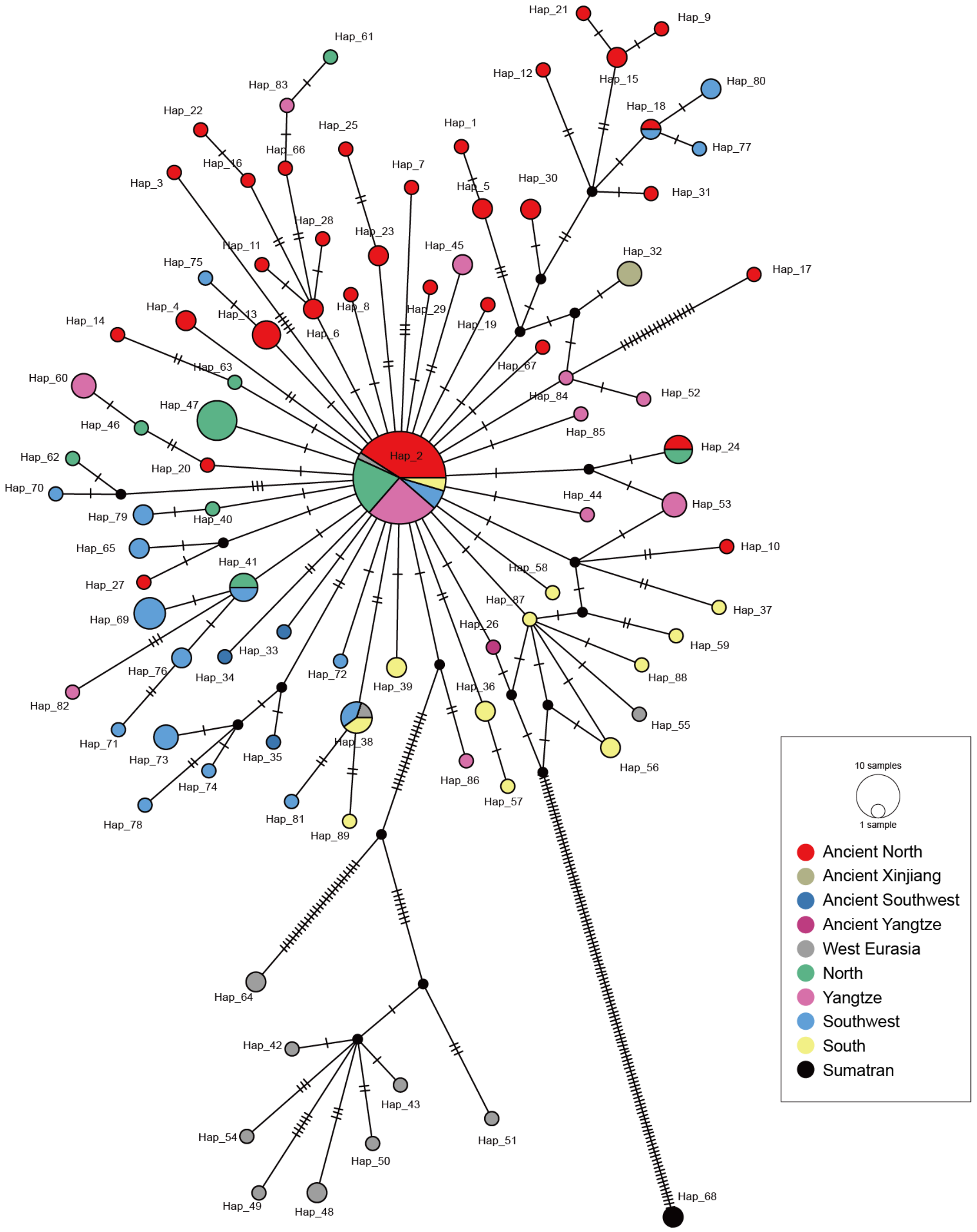

Supplementary fig. S2. Median-Joining haplotype network of mitochondrial genomes. The network was constructed using the same sample set as the ML tree. The size of dots shows the number of samples in each of the haplotype, and each short line indicates one base difference between the haplotypes.

Section 3: Autosomal phylogenetic analysis

In the Autosomal Neighbor-joining phylogenetic tree (autosomal NJ tree) (supplementary fig. S3), the populations from Eastern and Western Eurasia formed two distinct clades, with the Jicha wild boar (Figs. 1A and 1B, supplementary table S1A) positioned basal to both of the clades (supplementary fig. S3). The ancient wild boars in Xinjiang (from Dalete and Halehaxite), along with some modern domestic pigs from Northern China (Nanyang and Min breeds) (Fig. 1A, supplementary table S1C), clustered together with samples from Western Eurasia (supplementary fig. S3). This indicated that the wild boars from Xinjiang were more closely related to the populations in Western Eurasia, and some modern breeds in Northern China have been significantly influenced by the gene flow of Western Eurasian populations.

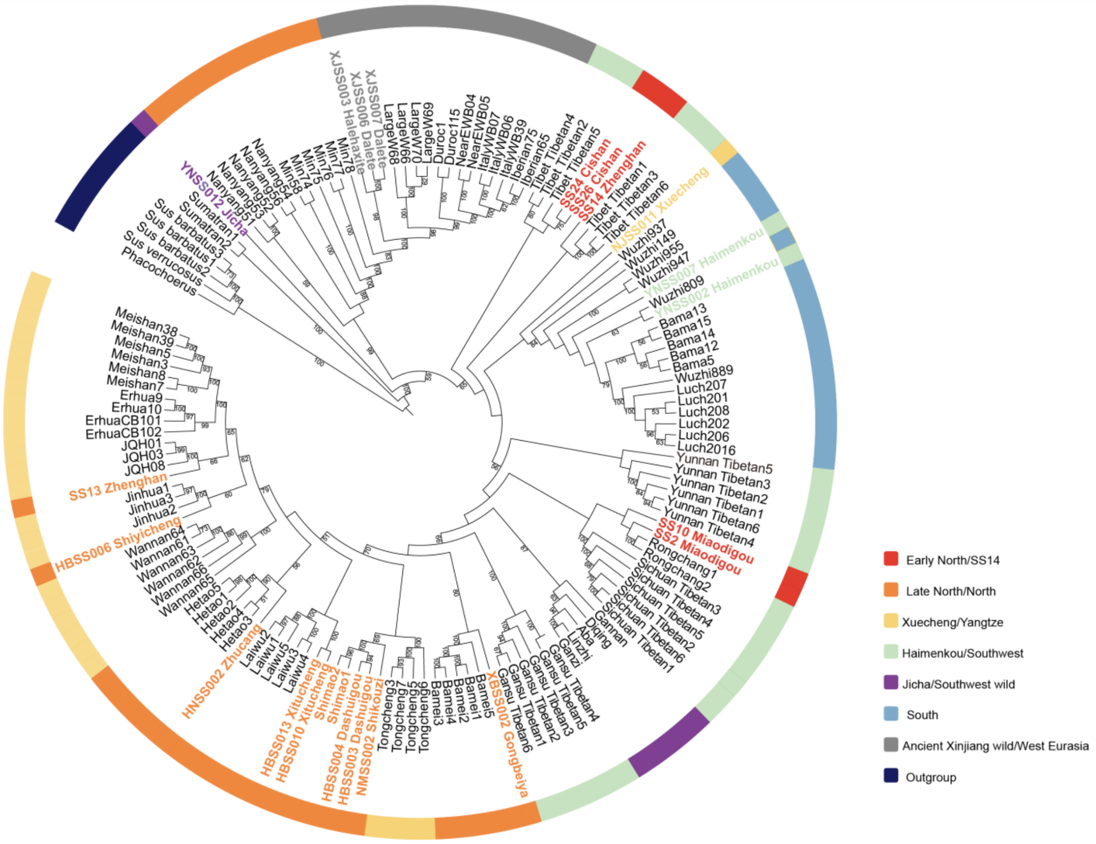

Supplementary fig. S3. Autosomal Neighbor-Joining phylogenetic tree of Eurasian pigs and wild boars. The tree was constructed by estimating the genetic distance between samples on 1262K SNPs with 100 bootstrap replicates. Colors of different groups have been described in the legend, consistent with Fig. 1. Tip labels of ancient samples have been

colored according to their respective groups to distinguish them from modern samples. The nodes supported by over 50% bootstrap replicates were shown with the support value.

Within the Eastern Eurasia clade, modern samples primarily diverged according to their geographical locations sequentially from the southwest-south (pigs and wild boars from Southwestern China and Southern China) to north-east (pigs from Northern China and the Yangtze River Basin) (supplementary fig. S3). This division aligned well with the results of PCA (fig. 1C) and was similar to the findings of published whole-genome analyses of modern domestic pigs in East Asia (Peng et al. 2022).

For the ancient samples, there was also a divergence between Early North and Late North. The individuals assigned to Early North together with a domestic pig from the ancient Yangtze River Basin (NJSSS011 from Xuecheng) (Figs. 1A and 1B, supplementary table S1A) occupied relatively basal positions and were genetically closely related to samples from the southwest-south (supplementary fig. S3). The samples belonging to Late North were closely nested within the domestic pigs from the north-east group. Additionally, samples from the Miaodigou site (SS2 and SS10, ~3,000 BCE) (Figs. 1A and 1B, supplementary table S1A) that were assigned to Early North according to PCA and ADMIXTURE analysis (Figs. 1C and 1D), showed a closer relationship to Late North and north-east modern pigs, suggesting that these samples might represent a transitional stage between Early North and Late North (supplementary fig. S3). Furthermore, the ancient domestic pigs in Southwestern China (YNSS002 and YNSS007 from Haimenkou) (Figs. 1A and 1B, supplementary table S1A) have clustered with the breeds (Bama, Luchuan, Wuzhishan) (Fig. 1A, supplementary table S1C) in Southern China underlying the affinity between the pig population in ancient Southwestern China and modern Southern China (supplementary fig. S3).

Section 4: Unsupervised population clustering analyses

According to the results of ADMIXTURE (Alexander et al. 2009) analysis (supplementary fig. S4A), Early North, Late North, who have shown clear divergence in the PCA (Fig. 1C), also exhibited differences regarding ancestral components. The Miaodigou pigs (SS2 and SS10) assigned to Early North, displayed less complex ancestral components compared to other members of the group. They seemed to occupy a transitional position between Early North and Late North. The ancient domestic pig from the Yangtze River Basin (NJSS011 from Xuecheng) shared identical ancestries with the Early North. These observed patterns were consistent from K=5 to K=7 (supplementary fig. S4B). The ancestries of the ancient wild boar from Southwestern China (YNSS012 from Jicha), in comparison to the ancient Southwest domestic pigs in the Haimenkou Site, possessed more basal ancestries akin to those of the Sumatran wild boar. When K=7, the Jicha wild boar exhibited a completely identical ancestry with Sumatran wild boar, reflecting its basal position to the Eurasia populations as revealed by the NJ tree (supplementary fig. S3). The samples from Ancient Xinjiang exhibited ancestries from both Eastern and Western Eurasia from K=5 to K=6, with a dominance of the Western Eurasia components. And when K=7, they showed an entirely similar component with some of the Western Eurasia individuals.

Modern samples from Eastern and Western Eurasia always exhibited distinct ancestries (supplementary fig. S4A). Within the Eastern Eurasia population, Chinese samples clustered according to specific geographical locations: Northern China (North), Yangtze River Basin (Yangtze), Southwestern China (Southwest), and Southern China (South). However, modern pigs from China were characterized by a significant proportion of Western Eurasian ancestries. This suggested extensive introgression from modern Western Eurasian pigs to Northern China. Modern Southwestern pigs and wild boars displayed ancestries differing from a local ancient wild boar in Jicha, but were more similar to ancient Northern China. This indicated that the populations in the region could have been largely influenced by Northern pigs, including the wild boars (supplementary fig. S4A).

When K=5, it gave the lowest value of cross-validation error (supplementary fig. S4B). Thus, we mainly showed the results of K=5 in Fig. 1D of the main text.

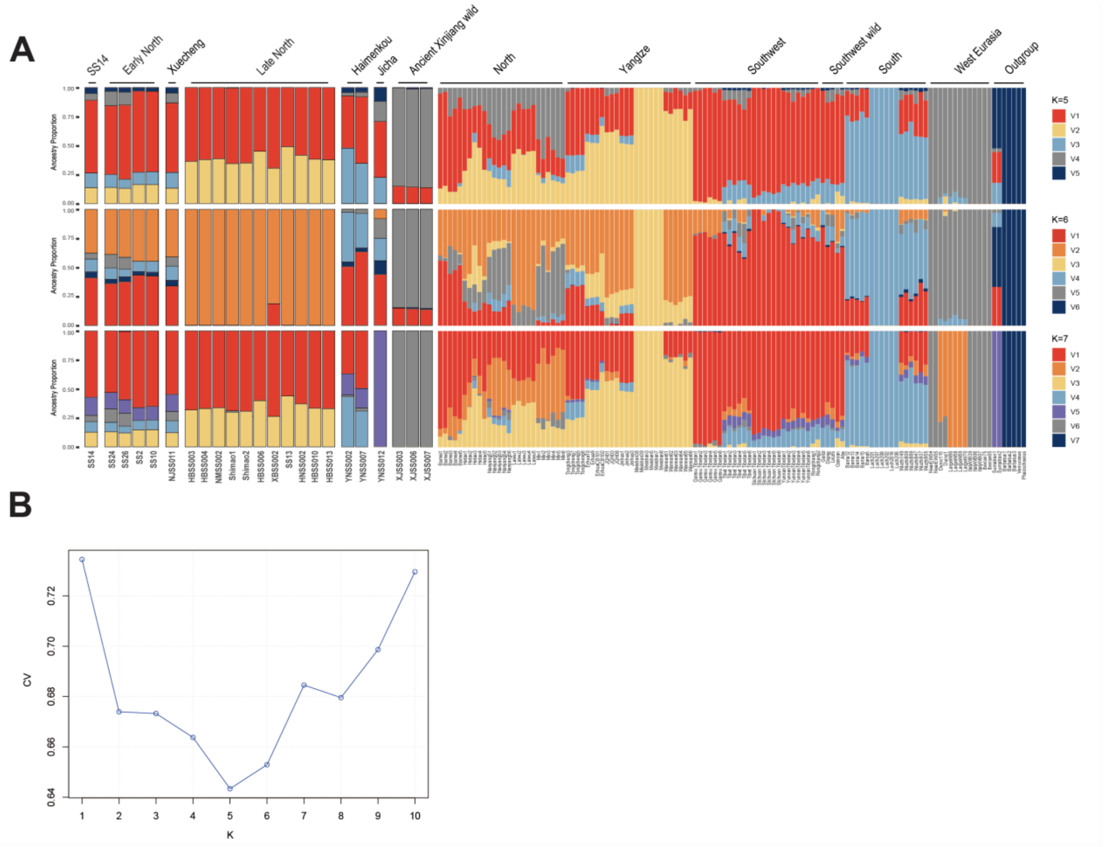

Supplementary fig. S4. ADMIXTURE analysis. (A) The results of ADMIXTURE analysis from K=5 to K=7. (B) Values of cross-validation (CV) error from K=1 to K=10. It gives the lowest value when K=5. The result when K=5 has also been shown in Fig. 1D of the main text.

We also performed DyStruct (Joseph and Pe'er 2019) (supplementary fig. S5) and Struct-f_4 (Librado and Orlando 2022) (supplementary fig. S6) to validate the population clustering patterns obtained through ADMIXTURE analysis. These two analyses mostly consolidated the findings of the ADMIXTURE (supplementary fig. S4A). However, different from the ADMIXTURE (supplementary fig. S4A) results, the ancient wild boar from Northern China (SS14 from Zheng and Han City) (Figs. 1A and 1B, supplementary table S1A) showed different ancestral components with other Early North members from K=6 in DyStruct analysis, indicating potential genetic differences between wild boar and domestic pigs in a minor degree. The pattern of Miaodigou pigs as a transitional form was less prominent in both DyStruct and Struct-f_4. The discrepancies among those analyses were likely caused by the algorithms behind them.

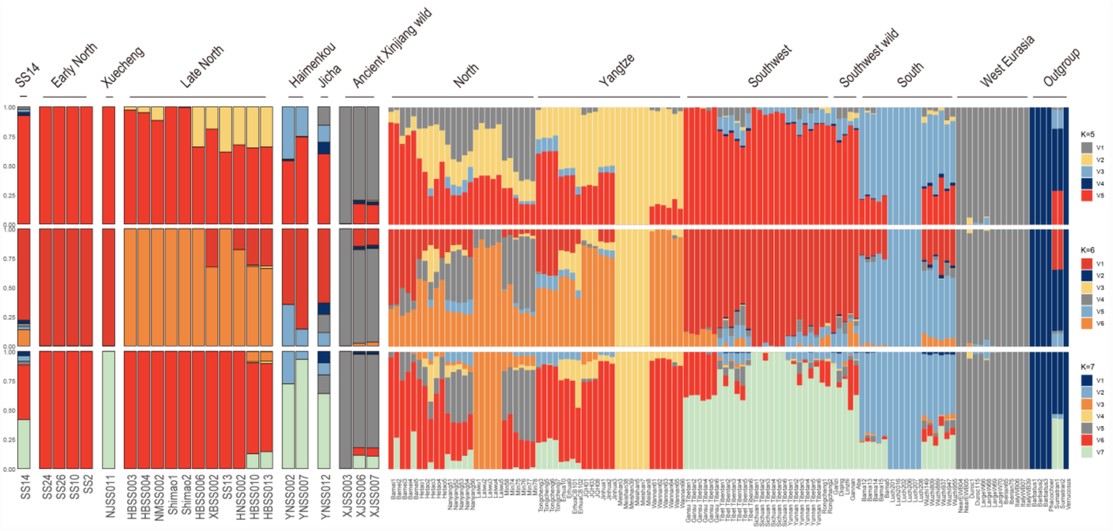

Supplementary fig. S5. The results of DyStruct analysis from K=5 to K=7.

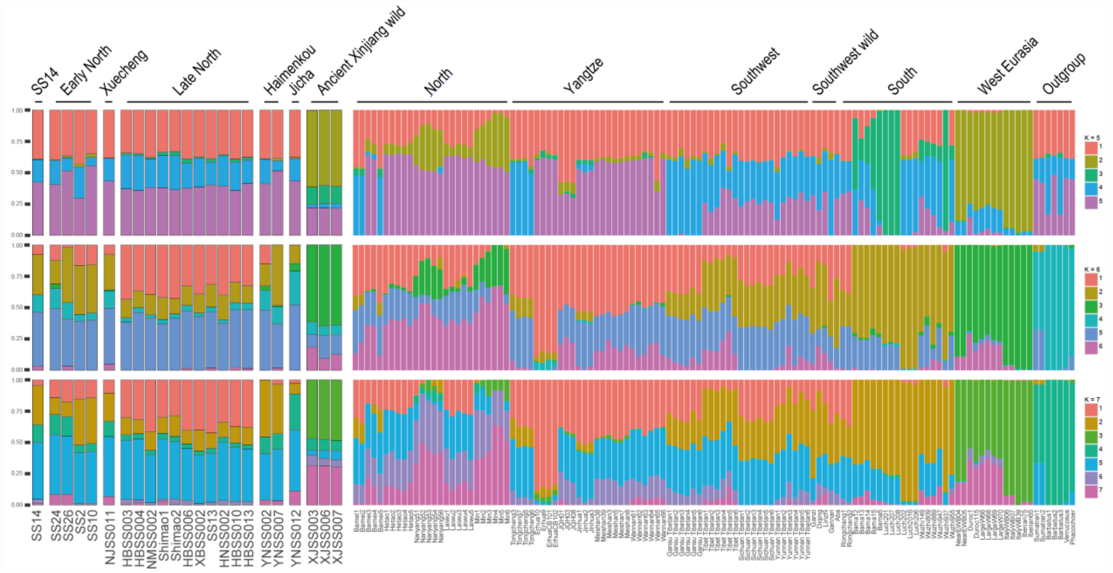

Supplementary fig. S6. The results of Struct-f_4 analysis from K=5 to K=7.

Section 5: Outgroup f_3 and f_4-statistics by archaeological sites

To validate the rationality of the Early North and Late North groupings, we conducted sites-based outgroup f_3-statistics and f_4-statistics analyses (Patterson et al. 2006; Patterson et al. 2012). With f_3(Site1, Site2; Sumatran), it has shown that domestic pigs from Late North sites displayed a higher genetic affinity compared to other groups, supporting the coherence of this grouping (supplementary fig. S7, supplementary tables S2A and S2B). In contrast, while sites within Early North exhibited certain similarities, they did not cluster as closely as those in Late North. We considered that it could be attributed to the early stage of domestication, during which pig populations still retained genetic heterogeneity as their wild ancestors. The Xuecheng individual (NJSS011) from the lower Yangtze River showed a more distant relationship with Northern groups in this analysis. This might suggest an independent origin from wild boars closely related to the boar population in Northern China, or an early introduction of pigs from Northern China, with additional local genetic contribution from the lower Yangtze River Basin.

Notably, Miaodigou who clustered with Early North in PCA (Fig. 1C), and unsupervised population clustering analyses (supplementary figs. S4 - S6) showed a higher affinity with Late North sites than with other Early North members as revealed by the population combination of f_4 (Sumatran, Miaodigou, Cishan/SS14, Late North sites) (supplementary table S2B). It was likely because Miaodigou pigs lived temporally closer to the period of Late North pigs. This has further consolidated its transitional position between Early North and Late North and implied the population continuity of ancient domestic pigs in Northern China since 6,000 BCE.

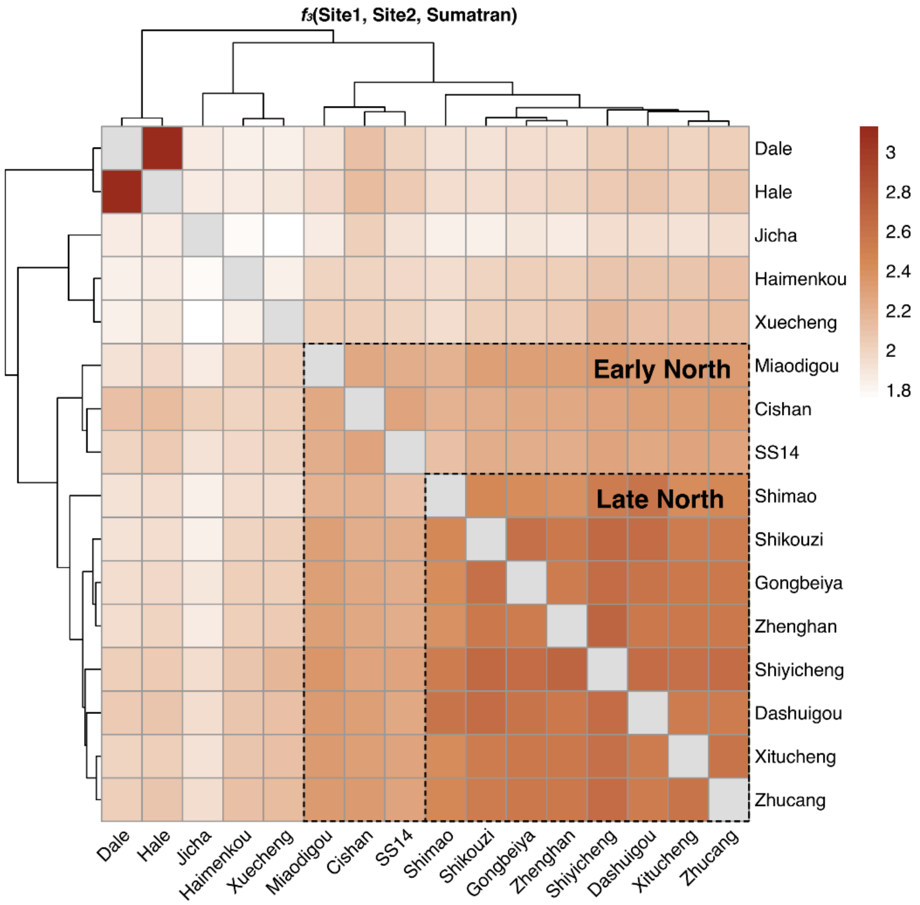

Supplementary fig. S7. The heatmap of pairwise outgroup f_3-statistics between ancient samples from different archaeological sites (supplementary table S2A). The group names of ancient samples from Northern China were labeled on their corresponding clusters.

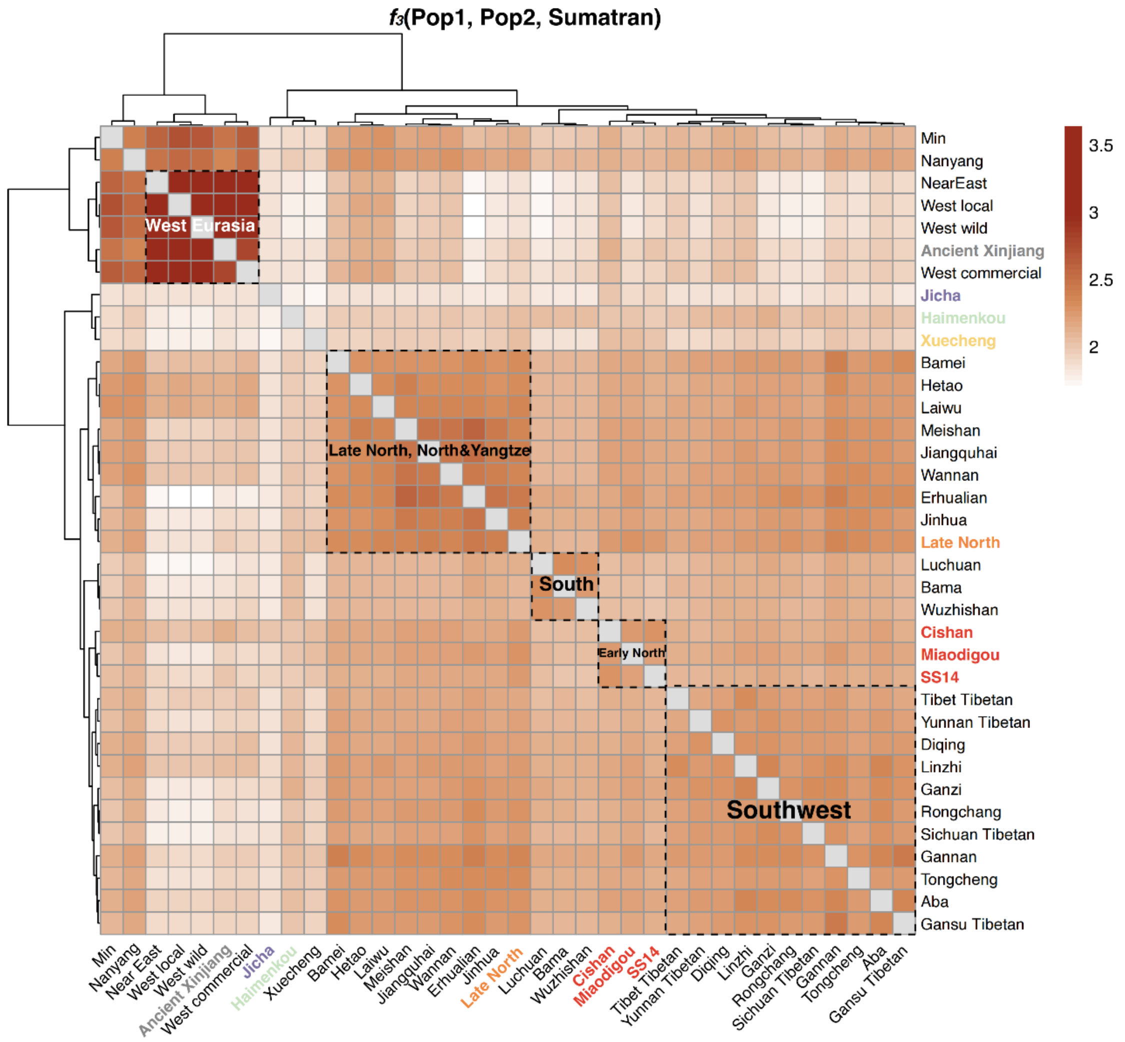

Supplementary fig. S8. The heatmap of pairwise outgroup f_3-statistics between ancient and modern groups or populations (supplementary table S2C). Ancient domestic pigs from Northern China post 2,000 CE were grouped (Late North). Colors of ancient populations or groups has been set to the same as Fig. 1 to distinguish them from modern populations.

Section 6: Run of Homozygosity

In ancient samples, our analysis indicated a notable accumulation of ROH segments (Ringbauer et al. 2021) in the later Late North population compared to the earlier Early North, with both an increase in long ROH segments (>12 cM) and short segments (4-12 cM) (Fig. 2C). This pattern suggested a possible reduction in population size among later Northern domestic pigs. Additionally, two individuals exhibited clear signs of inbreeding (with total ROH length for segments over 20 cM exceeding 50 cM): SS2 from Early North (Miaodigou site, 2,935-2,875 cal BCE) and NMSS002 from Late North (Shikouzi site, 1,421 - 1,262 cal BCE) (Fig. 2C and supplementary fig. S9, supplementary tables S1A and S1B). Both population size reduction and inbreeding signal pointed to the possibility of a bottleneck effect and artificial breeding during intensified human management in later Northern domestic pigs (Fig. 2C, supplementary fig. S9). Previous zooarchaeological and isotopic evidence has similarly proposed a shift in pig husbandry practices in the Central Plains of Northern China from free-range to captive management around 6,000-5,000 years ago, which aligned with our findings (Dong and Yuan 2020; Zhang et al. 2021; Yang et al. 2022; You et al. 2024).

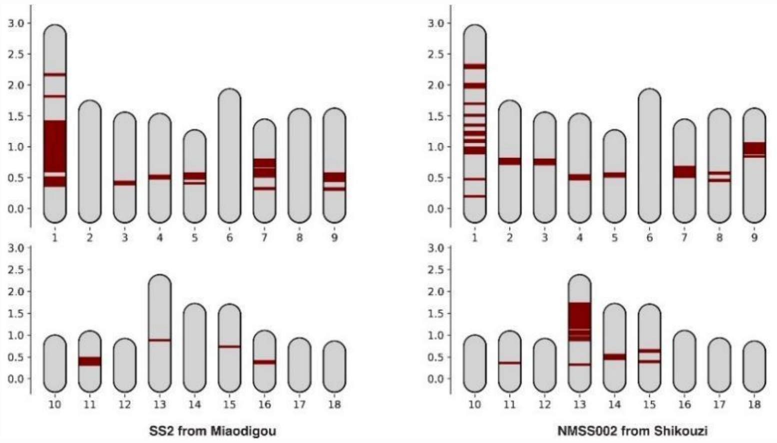

Supplementary fig. S9. The regions containing ROH (≥4cM) among different chromosomes of SS2 (left) and NMSS002 (right). The two individuals have been identified with accumulated long ROH (Fig. 2C) which might be caused by recent inbreeding.

Wild boars, such as SS14 and YNSS012, showed less ROH accumulation, as expected. The Xinjiang wild boars exhibited higher ROH levels compared to SS14 and YNSS012, possibly reflecting a smaller effective population size. The ancient

Haimenkou domestic pigs showed ROH levels comparable to Late North, potentially suggesting a similar local population size (Fig. 2C, supplementary fig. S9).

In addition to ancient samples, we also conducted ROH analysis on modern samples for comparison, using both hapROH (Ringbauer et al. 2021) and PLINK v1.9 (Purcell et al. 2007). The hapROH results indicated significant variation in total ROH lengths across modern domestic pig breeds in different regions. Generally. Individuals with high ROH accumulations (exceeding 900 cM) were commonly found in the breeds across China, with pigs in the South showing relatively lower ROH levels (supplementary fig. S10). The phenomenon could be attributed to full-sibling mating (such as a sibling or parent-offspring pairing) as a method which is often used in modern pig breeding to maintain a highly uniformed genetic background. When we restricted to short ROH fragments between 4-8 cM, we found that the Chinese breed pigs carried less ROH compared to European commercial breeds like Large White, indicating a larger effective population size of Chinese local breeds (supplementary fig. S10). The results from PLINK analysis were highly consistent with hapROH in terms of overall trends and total ROH accumulation (supplementary fig. S10).

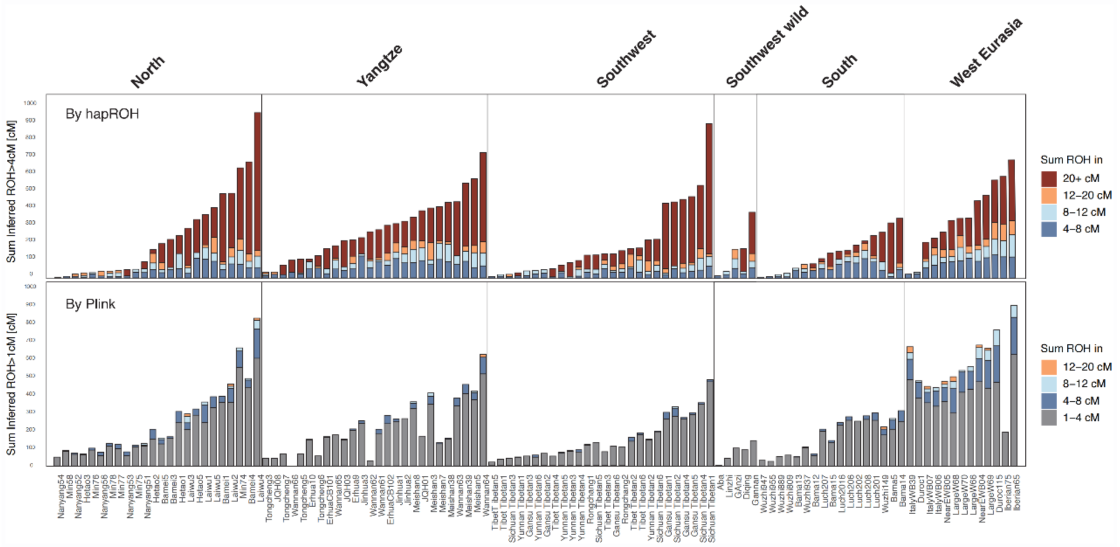

Supplementary fig. S10. The estimated ROH of modern samples using hapROH (top) and PLINK (bottom).

We also estimated the effective population sizes of Northern pigs through time. The N_e of Early North pigs was first estimated combining the early domestic pig and wild boar individuals, and then separately for the wild boar (SS14) and domestic pig (SS10) individuals. Both the results calculated using combined Early North group and SS10 were significantly higher than the Late North population. However, it was worth noting that the resulting confidence intervals with only one individual were excessively wide (Table S3).

Table S3. Effective population sizes of North groups estimated using hapROH

Population	Individual N*	Coeff (2N)	CI:0.025	CI: 0.975	N_e
**Early North**	2	1337	753	2688	668
**Late North**	8	282	242	333	141
**SS14**	1	2246	-324	4816	1123
**SS10**	1	944	226	1662	472

* Two samples with recent inbreeding signals were excluded from the analysis.

Section 7: Admixture modeling using qpAdm and qpGraph

Samples included in the analyses and their corresponding populations have been shown in supplementary table S1A.

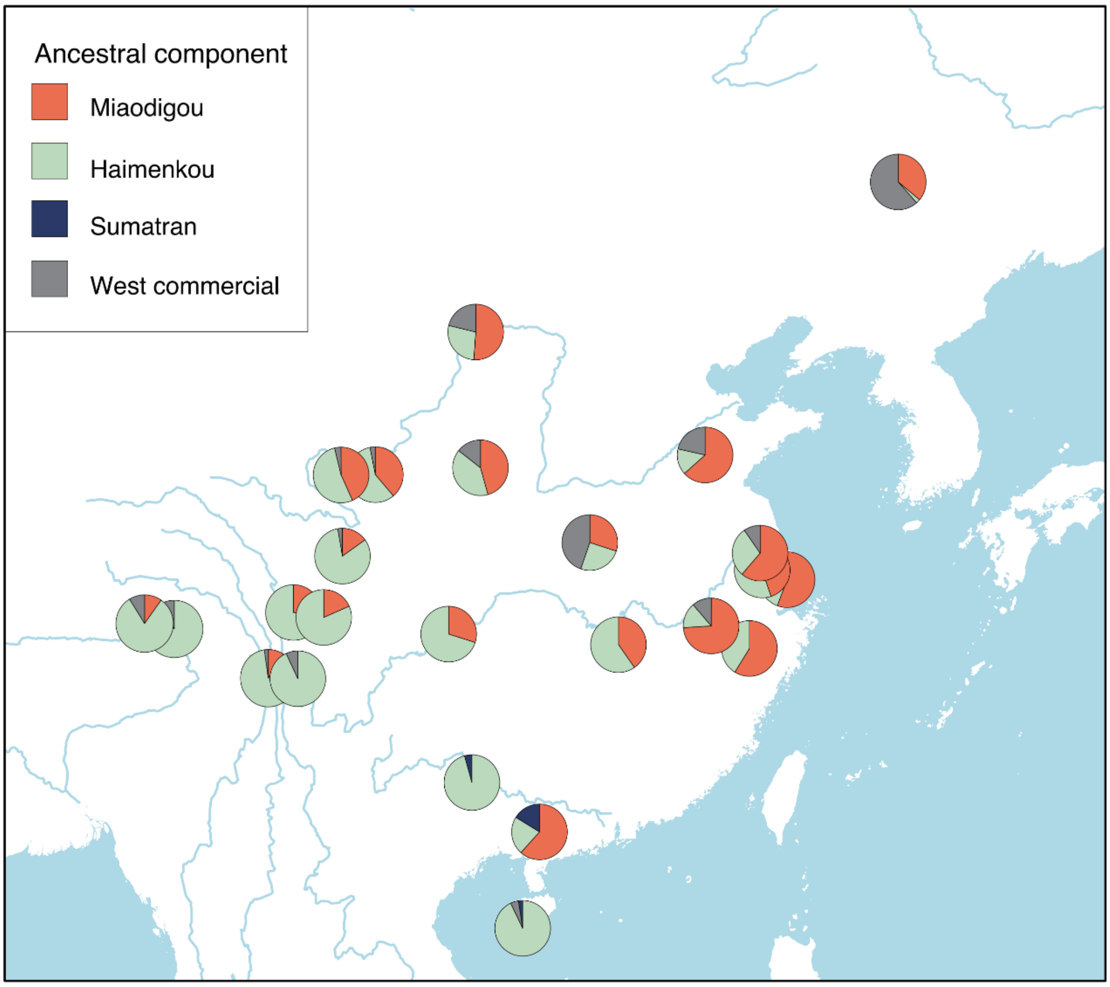

Supplementary fig. S11. The ancestry modelling of modern populations with qpAdm (supplementary table S4A).

Miaodigou, Haimenkou, Sumatran and West commercial were used to represent ancient Northern pigs, ancient Southwestern pigs, undescribed Southern wild boars, and Western Eurasian pigs. Pie charts showed the estimated proportion of each ancestry. The results using Late North to represent ancient Northern pigs has been shown in Fig. 3C.

The initial qpGraph model (Patterson et al. 2006; Patterson et al. 2012) (supplementary fig. S12) based on predefined phylogenetic relationships according to the Autosomal NJ tree aligned with established phylogenetic relationships and showed a good fit (Z= -2.262). The Sumatran wild boar was positioned ancestral to the Eurasian clade, followed by the divergence of Western Eurasian and Asian

populations. Moreover, no significant gene flow was observed between the four populations, and Early North appears as the direct ancestral population of Late North.

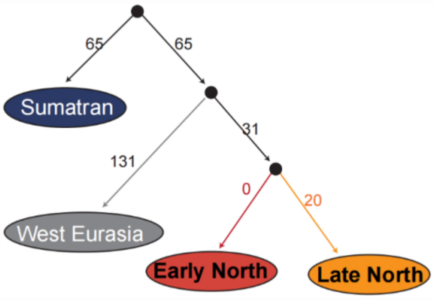

Supplementary fig. S12. Admixture modeling using four populations (Z: -2.262).

Building upon the initial 4-population model, we added ancient domestic pigs from the Haimenkou site in Southwestern China (supplementary fig. S13). The model indicated that the Haimenkou pigs can be modeled as a derived branch of the ancient Northern Chinese population (a sister group to Late North), mixed with an unknown population that likely represented local wild boars in Southwestern China at the base of the Eurasian clade (Z= -2.26). This suggested that Northern Chinese domestic pigs migrated to Southwestern China, and mixed with local wild boars.

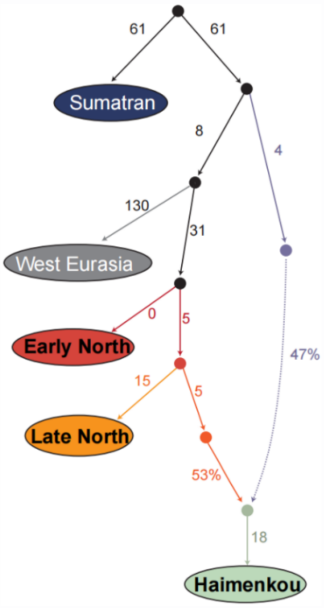

Supplementary fig. S13. Admixture modeling using five populations (Z: -2.26).

After analyzing the evolutionary relationships of ancient pigs, we expanded the 5-population model by adding modern populations from different geographic regions. This allowed us to investigate the genetic contributions of ancient populations to modern Chinese domestic pigs (supplementary figs. S14-S17).

Autosomal NJ tree and *f*-statistics indicated a close genetic relationship between modern pigs from Northern China, the Yangtze River, and the Late North populations (supplementary fig. S3, supplementary tables S2C and S2G). To further explore this relationship, we incorporated the modern North and Yangtze populations into the existing 5-population model. First, we fitted both groups using a 2-way admixture model (supplementary figs. S14A and 14B). The results showed that the best-fitting 2-way models for both regions had the same topology, with both modeled as a mixture of a Late North-related lineage and the Western Eurasian population. The proportion of Western Eurasian ancestries differed slightly between North and Yangtze, with North pigs showing more ancestries of Western Eurasia compared to Yangtze pigs (supplementary figs. S14A and S14B). Given the high similarity in topology, we merged the two populations into one group, referred to as “North and Yangtze” (supplementary fig. S14C). However, the |Z| for the 2-way admixture models consistently showed high values, both before and after merging (Z = 6.707, 3.398 and 5.342, respectively). F_4-statistics suggested that these 2-way models underestimated the genetic relationship between the North and Yangtze pigs and the ancient domestic pigs from the Haimenkou site, consistent with the Haimenkou ancestry found in some North and Yangtze River modern pigs in qpAdm analyses (Fig. 3A, supplementary fig. S11).

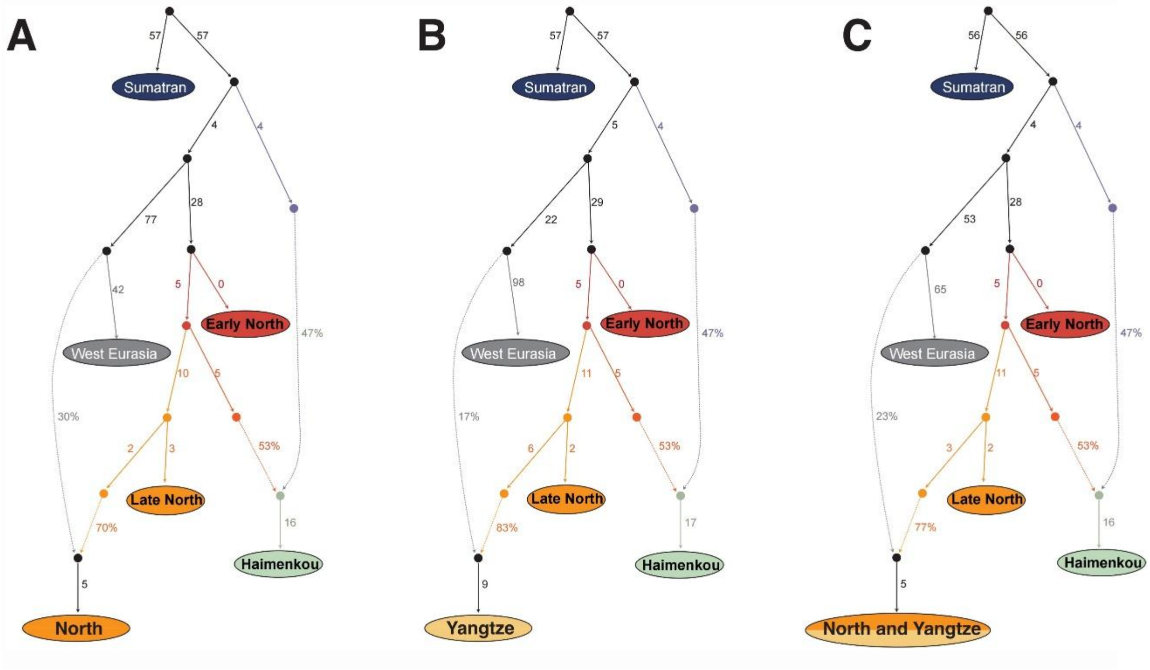

Supplementary fig. S14. Two-way admixture modeling for modern domestic pigs. (A) Admixture graph of modern domestic pigs from Northern China (Z=6.707). (B) Admixture modeling for modern domestic pigs from the Yangtze River Basin (Z=3.398). (C) Combined admixture model for modern domestic pigs from both Northern China and the Yangtze River Basin, with a Z-score of 5.342.

We subsequently applied a 3-way admixture model to fit the modern pigs from the North, Yangtze and both combined (supplementary fig. S15). The results indicated that both modern populations can be modeled as a three-way admixture of the lineages related to Late North, Haimenkou, and Western Eurasian population (Z = -2.212 for North, -2.194 for Yangtze and -2.228 for both combined).

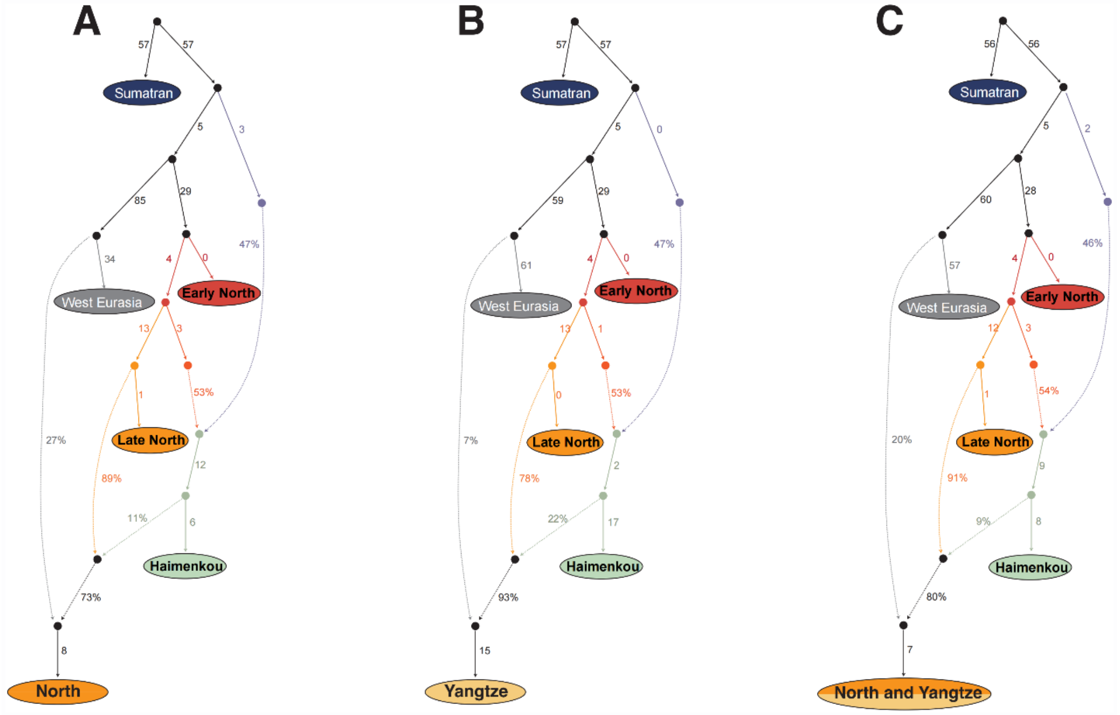

Supplementary fig. S15. Three-way admixture modeling for modern domestic pigs, all models demonstrating a well-fitted scenario with |Z|≤3. (A) Admixture graph of modern domestic pigs from Northern China (Z=-2.212). (B) Admixture graph of modern domestic pigs from the Yangtze River Basin (Z=-2.194). (C) Combined admixture model for modern domestic pigs from both Northern China and the Yangtze River Basin (Z=-2.228).

Based on the results, we inferred that the genetic composition of modern pigs in Northern China and the Yangtze River Basin was largely influenced by the Late North-related population, with additional contributions from Southwestern domestic

pigs (supplementary fig. S15). The geographic connection between Northern China and the Yangtze likely resulted in frequent gene flows between the two populations, subsequently leading to their high genetic similarity. However, the pig population from Northern China has received a higher proportion of Western Eurasian ancestries compared to the Yangtze population (supplementary figs. S15A and S15B).

We then incorporated pigs from the Southwestern and Southern China into the 5-population model. The results showed that modern pigs from Southwestern China shared a high degree of genetic similarity with ancient Northern pigs, which could be modeled as a mixture of a sister lineage to Late North and an unknown wild boar population basal to Eurasian pigs. (Z= -2.180; supplementary fig. S16).

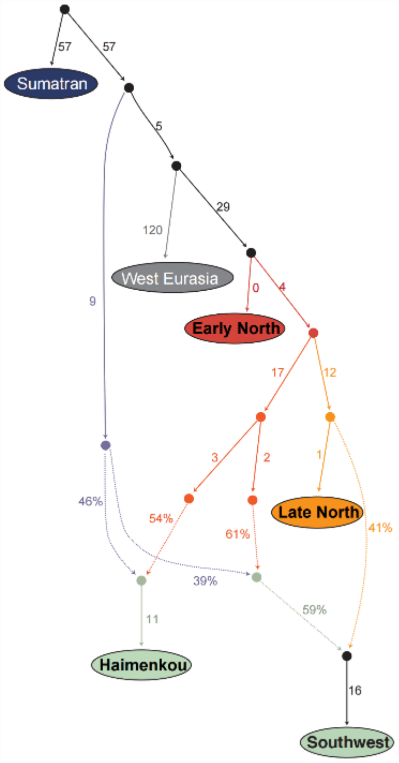

Supplementary fig. S16. Admixture modeling for modern domestic pigs from Southwestern China (Z=-2.180).

Modern pigs from Southern China were modeled as a mixture of Haimenkou, the ancient domestic pigs from Southwestern China and a sister lineage related to Late North (Z= -2.992; supplementary fig. S17). Through the results, we inferred that the ancient Southwestern pigs further migrated southward, reaching Southern China and mixing with an unknown Southern wild boar population.

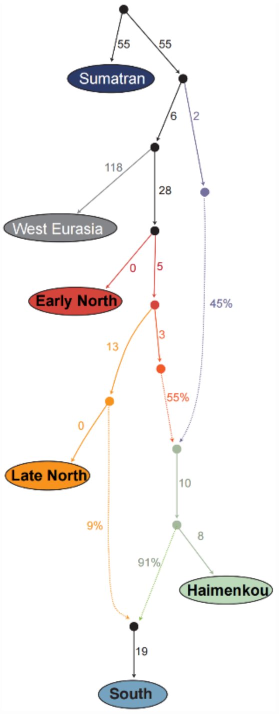

Supplementary fig. S17. Admixture modeling for modern domestic pigs from Southern China (Z=-2.992).

After modeling the modern pigs from individual regions, we integrated all ancient and modern populations to create an admixture graph of Chinese domestic pigs (Z= 3.998; Fig. 3A). The graph was too complex to be fitted within the threshold ($|Z| \leq 3$), by incorporating all the populations together. We therefore chose a slightly more relaxed threshold of $|Z| \leq 4$ for the full model incorporating all populations.

Section 8: The evolution of black coloration in Chinese domestic pigs

The black coat phenotype in Chinese domestic pigs is caused by a single nucleotide variant (Sscrofa11 Chr6:181883 A>G) in the melanocortin 1 receptor gene (*MC1R*) (Fang et al. 2009).

Among the ancient and modern samples used in this study, 118 individuals had sequencing reads that covered this site. Samples and their corresponding read coverage details have been provided in supplementary Table S5A. We calculated allele frequencies in different populations using maximum likelihood estimation based on REF/ALT read counts (Mathieson et al. 2015). For each individual i, with j_i reads mapped to the reference allele and k_i reads mapped to the alternate allele, the likelihood function for allele frequency p is:

$$\mathrm{L(p)} = \prod_{i=1}^{N} \left(C_{k_i+j_i}^{k_i}\, p^{k_i} (1-p)^{j_i} \right)$$

Using the formula above, we calculated the maximum likelihood estimates of allele frequency for each ancient and modern group. We determined the 95% confidence interval (CI) based on a log-likelihood value of lnL(p) = -1.92. Allele frequency estimates were made for six modern Eurasian populations and five ancient Chinese groups; as a wild boar, SS14 was analyzed separately. The likelihood curves for each group at the *MC1R* variant site has been shown in supplementary fig. S18 and supplementary table S5B.

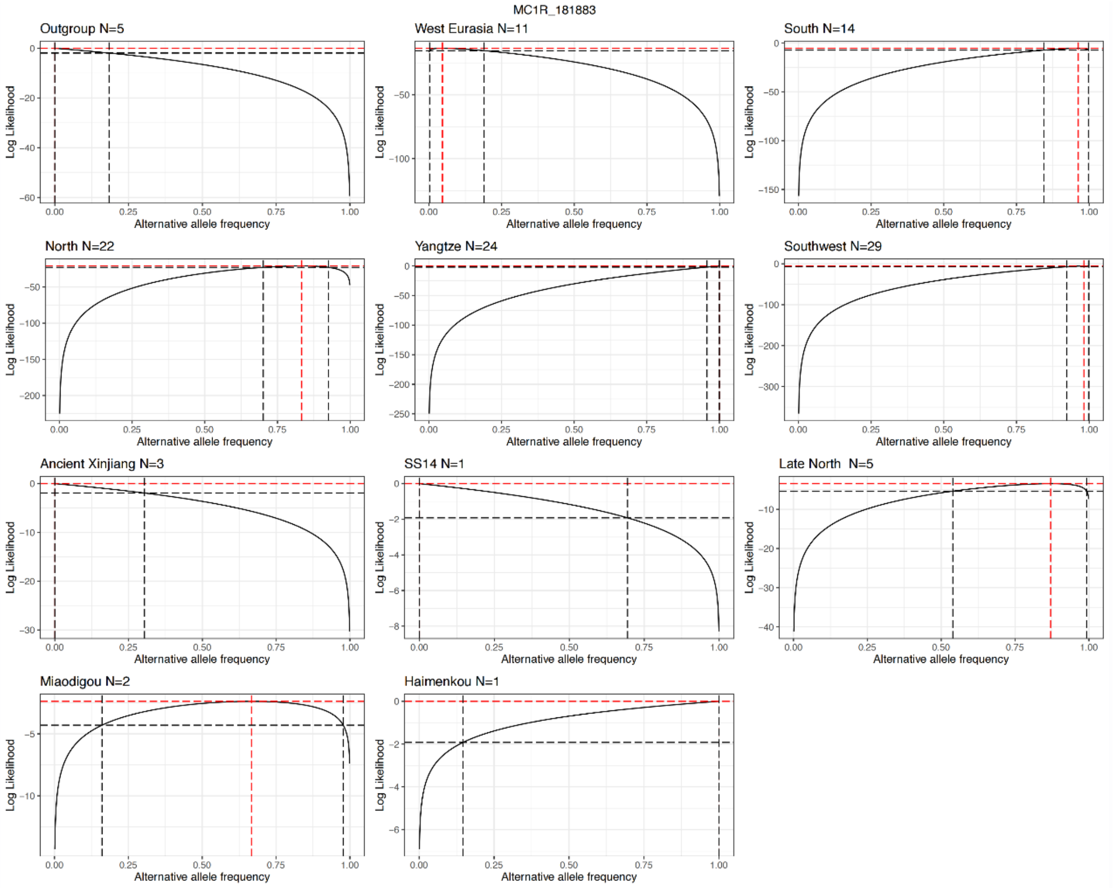

Supplementary fig. S18. **The likelihood curves of the alternative allele frequency (Sscrofa11 Chr6:181883 G>A) in *MC1R* gene for each population of pig/wild boar (Fig. 4 and supplementary table S5B)**

We also conducted a haplotype analysis on the *MC1R* gene. To minimize the impact of missing data, we first used GLIMPSE v1.1.1 (Rubinacci et al. 2021) to impute chromosome 6 (where the *MC1R* gene is located) for all ancient and modern samples using SWIM reference panel (Ding et al. 2023). Following the imputation, despite the network analysis (Fig. 4B, supplementary fig. S20, supplementary table S5D), we have also drawn a heatmap showing the clustering of haplotypes of *MC1R* and 25 kb flanking regions using all samples. In the heatmap (supplementary fig. S19), we observed a clear haplotype distinction among domestic pigs from Western and Eastern Eurasia and wild boar samples. From the *MC1R* region onward, Asian domestic pigs displayed a distinct pattern of haplotype linkage different from those in Western Eurasia and wild boars. At least one haplotype from the ancient domestic pigs as early as 3,300 to 3,000 BCE (pigs from Miaodigou) was within the clade of Eastern Eurasian domestic pigs (Fig. 4B, supplementary fig. S20). The results

indicated the unique genetic mechanism of the black coloration in Chinese domestic pigs compared to their European counterparts, and a potential single origin of the black-coat phenotype that likely first occurred in Northern China.

Additionally, following the imputation of position 181,883 within the *MC1R* gene, we directly calculated allele frequencies at this position for each group while excluding samples with GP < 0.95 (Fig. 4A, supplementary table S5C).

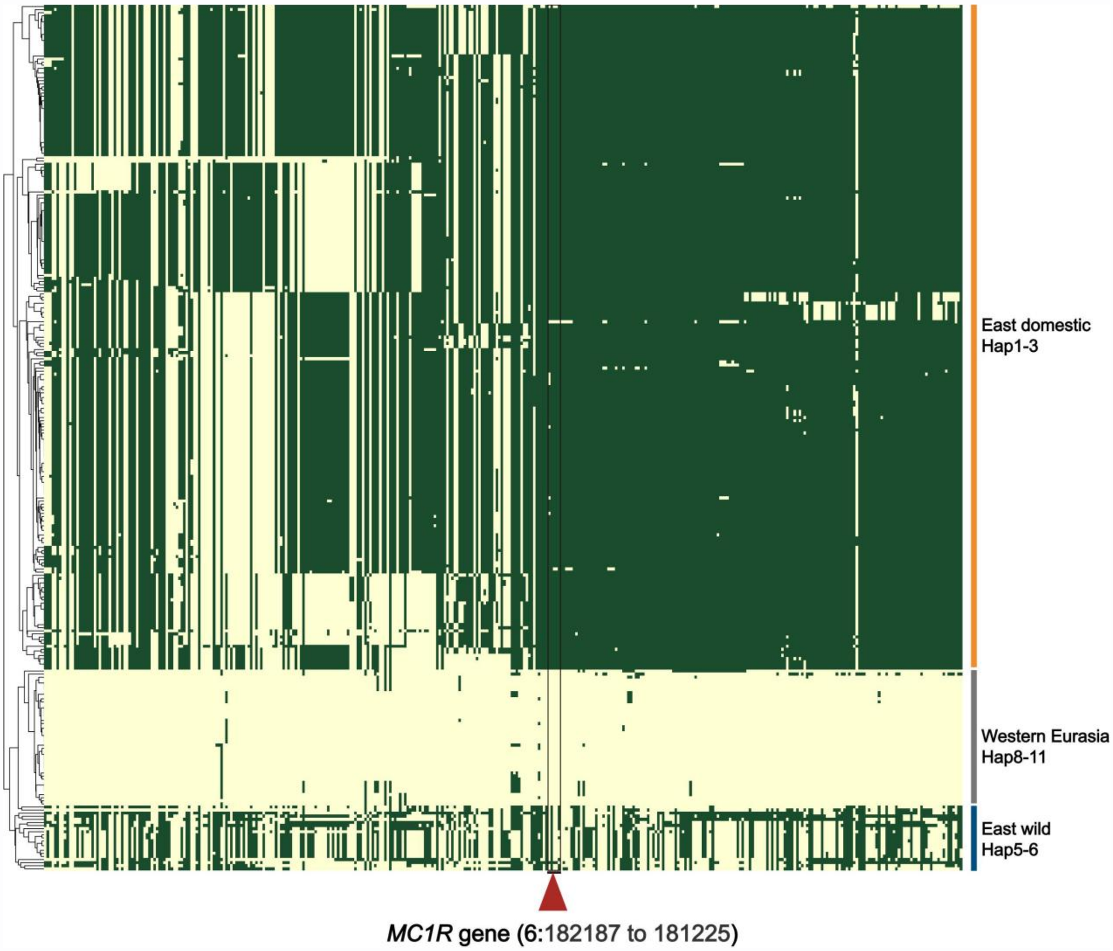

Supplementary fig. S19. Haplotype heatmap of 25 kb regions upstream and downstream of the *MC1R* gene (6:156225-207187) using imputed data. The location of the MC1R gene has been indicated by a dark red arrow. The *MC1R* gene haplotypes of the samples were labeled on the right side of the heatmap.

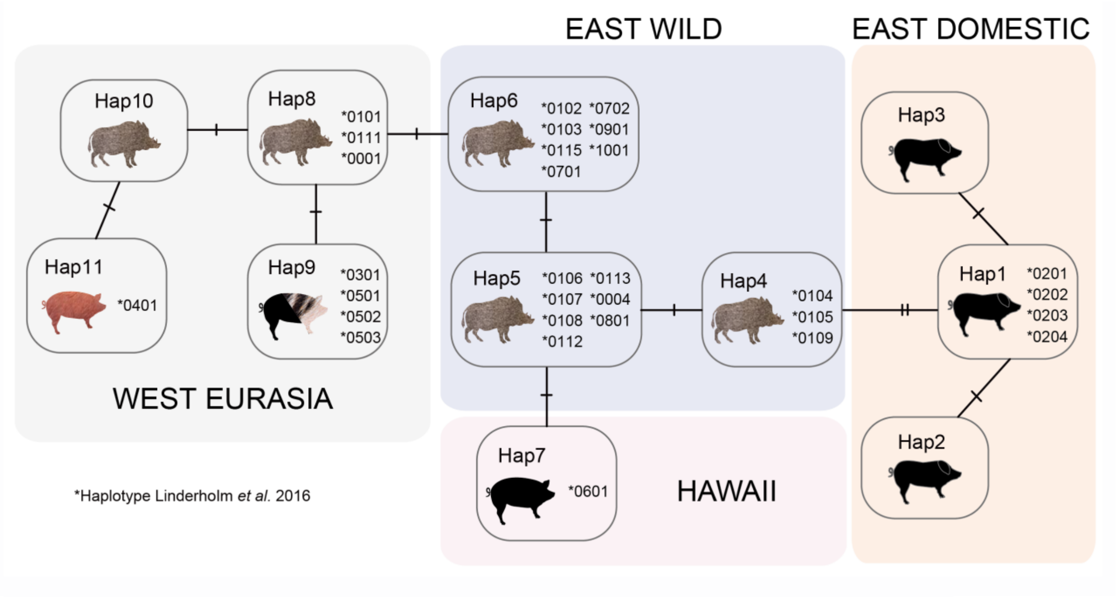

Supplementary fig. S20. Haplotype network of the *MC1R* gene and its correspondence with haplotypes reported in Linderholm *et al*. (Fig. 4B, supplementary table S5E). The network was constructed using the imputed and phased *MC1R* gene data. Haplotypes identified in this study has been shown alongside their corresponding haplotype names from Linderholm *et al.*(Linderholm et al. 2016).

Section 9: A description of the dispersal map, related to (Fig. 5)

Combining our findings with existing archaeological and ancient DNA research on East Asian human movements, we described the evolutionary history and migration routes of Chinese domestic pigs over the past 8,000 years.

In at least 6,000 BCE, pigs were domesticated in Northern China. The Early North pigs experienced a population bottleneck about 3,500 to 3,000 BCE, due to intensified domestication practices, resulting in decreased genetic diversity and recent inbreeding (Dong and Yuan 2020; Zhang et al. 2021; Yang et al. 2022; You et al. 2024) and gave rise to Late North pigs. Domestic pigs in Northern China formed a genetically continuous population spanning over 7,000 years, until the introduction of European domestic pigs from the 19th century significantly influenced their native genetic pool (Frantz et al. 2019).

Towards the Southwest, Northern pigs were introduced into Southwestern China accompanied by the arrival of Yellow River farmers to the Qinghai-Tibet Plateau likely from 3,500 BCE (Ren et al. 2020a; Wang et al. 2023). These pigs mixed with an undescribed wild boar population on the way and served as a primary ancestral component for modern Southwestern domestic pigs. The ancient Southwestern pigs probably continued to migrate southward, reaching Southern China and again received gene flow from another unknown pig or wild boar lineage in Southern China along with the human migration (Ren et al. 2020b; Tao et al. 2023; Wang et al. 2023).

Towards the Southeast, Northern pigs dispersed to the Yangtze River Basin, and formed the modern breeds in this region which share high genetic affinity with Late North pigs. The frequent interactions between domestic pigs from the two areas also reflected the frequent exchange and integration of human populations (Yang et al. 2020; C.-C. Wang et al. 2021).

A bidirectional diffusion between the pigs in Southwest and East China was then observed in the gene pool of modern pig breeds. Both migration waves contributed to the establishment of pig populations in Southern China, mirroring the genetic

connection seen in historical Southern China people with Northern and Southern East Asian ancestries (T. Wang et al. 2021; Tao et al. 2023).

Supplementary References

Alexander DH, Novembre J, Lange K. 2009. Fast model-based estimation of ancestry in unrelated individuals. *Genome research* 19:1655–1664.

Chen C, Zhang W, Liu K. 2014. The Archaeological Survey of the Xitucheng Site, Kangbao, Hebei Province. *Steppe Cultural Relics*:63–77.

Chen H, Huang M, Yang B, Wu Z, Deng Z, Hou Y, Ren J, Huang L. 2020. Introgression of Eastern Chinese and Southern Chinese haplotypes contributes to the improvement of fertility and immunity in European modern pigs. *Gigascience* 9:giaa014.

Ding R, Savegnago R, Liu J, Long N, Tan C, Cai G, Zhuang Z, Wu J, Yang M, Qiu Y. 2023. The SWine IMputation (SWIM) haplotype reference panel enables nucleotide resolution genetic mapping in pigs. *Communications Biology* 6:577.

Dong N, Yuan J. 2020. Rethinking pig domestication in China: regional trajectories in central China and the Lower Yangtze Valley. *Antiquity* 94:864–879.

Fang MeiYing FM, Larson G, Ribeiro HS, Li Ning LN, Andersson L. 2009. Contrasting mode of evolution at a coat color locus in wild and domestic pigs. *PLoS genetics* 5.1: e1000341.

Frantz LA, Haile J, Lin AT, Scheu A, Geörg C, Benecke N, Alexander M, Linderholm A, Mullin VE, Daly KG. 2019. Ancient pigs reveal a near-complete genomic turnover following their introduction to Europe. *Proceedings of the National Academy of Sciences* 116:17231–17238.

Frantz LA, Schraiber JG, Madsen O, Megens H-J, Cagan A, Bosse M, Paudel Y, Crooijmans RP, Larson G, Groenen MA. 2015. Evidence of long-term gene flow and selection during domestication from analyses of Eurasian wild and domestic pig genomes. *Nature genetics* 47:1141–1148.

Fu J, Li Yuniu, Hu C, Pan G, Yang X, He Q, Higham C, Li Yingfu. 2024. Southbound transmission of metallurgy: new excavations at Jicha in the Hengduan Mountains, Yunnan. *Antiquity*:1–8.

Han J. 2013. The Miaodigou Age and “early China.” *Chinese Archaeology* 13:163–168.

Yuan J, Flad RK. 2002. Pig domestication in ancient China. *Antiquity* 76:724–732.

Joseph TA, Pe’er I. 2019. Inference of population structure from time-series genotype data. *The American Journal of Human Genetics* 105:317–333.

Larson G, Liu R, Zhao X, Yuan J, Fuller D, Barton L, Dobney K, Fan Q, Gu Z, Liu X-H. 2010. Patterns of East Asian pig domestication, migration, and turnover revealed by modern and ancient DNA. *Proceedings of the National Academy of Sciences* 107:7686–7691.

Li Q, Guo J, Wu Z. 2020. Preliminary Study on the Animal Remains Excavated from the Shiyicheng Site in Shijiazhuang, Hebei Province. *Cultural Relics in Southern China* 1:160–166.

Li Y, Huang Z, Dang Z, Chen T, Cheng R. 2022. Animal resource exploitation in ancient cities on the middle section of the Silk Road during 10th～ 14th centuries ad: evidence from animal remains unearthed from the Dalete ancient city in 2017. *Quaternary Sciences*:51–62.

Librado P, Orlando L. 2022. Struct-f4: a Rcpp package for ancestry profile and population structure inference from f 4-statistics. *Bioinformatics* 38:2070–2071.

Linderholm A, Spencer D, Battista V, Frantz L, Barnett R, Fleischer RC, James HF, Duffy D, Sparks JP, Clements DR, et al. 2016. A novel *MC1R* allele for black coat colour reveals the Polynesian ancestry and hybridization patterns of Hawaiian feral pigs. *R. Soc. open sci.* 3:160304.

Liu Y, Li T, Fan W, Hou Y, Song H. 2024. The subsistence strategy transformation of the Yangshao culture (6900-4800BP) in the Guanzhong Area and Western Henan based on new faunal materials from the Miaodigou site. *Journal of Archaeological Science: Reports* 58:104725.

Ma S. 1978. The Ancient Capital City of Zheng and Han State in Xinzheng. *Zhongyuan Wenwu (Cultural Relics of the Central Plains)* 2:54–55.

Mathieson I, Lazaridis I, Rohland N, Mallick S, Patterson N, Roodenberg SA, Harney E, Stewardson K, Fernandes D, Novak M. 2015. Genome-wide patterns of selection in 230 ancient Eurasians. *Nature* 528:499–503.

Patterson N, Moorjani P, Luo Y, Mallick S, Rohland N, Zhan Y, Genschoreck T, Webster T, Reich D. 2012. Ancient admixture in human history. *Genetics* 192:1065–1093.

Patterson N, Price AL, Reich D. 2006. Population structure and eigenanalysis. *PLoS genetics* 2:e190.

Peng Y, Cai X, Wang Y, Liu Z, Zhao Y. 2022. Genome-wide analysis suggests multiple domestication events of Chinese local pigs. *Animal Genetics*. *Animal Genetics* 53:293-306.

Purcell S, Neale B, Todd-Brown K, Thomas L, Ferreira MA, Bender D, Maller J, Sklar P, De Bakker PI, Daly MJ. 2007. PLINK: a tool set for whole-genome association and population-based linkage analyses. *The American journal of human genetics* 81:559–575.

Ren L, Dong G, Liu F, d'Alpoim-Guedes J, Flad RK, Ma M, Li H, Yang Y, Liu Y, Zhang D. 2020a. Foraging and farming: archaeobotanical and zooarchaeological evidence for Neolithic exchange on the Tibetan Plateau. *Antiquity* 94:637–652.

Ren L, Dong G, Liu F, d'Alpoim-Guedes J, Flad RK, Ma M, Li H, Yang Y, Liu Y, Zhang D. 2020b. Foraging and farming: archaeobotanical and zooarchaeological evidence for Neolithic exchange on the Tibetan Plateau. *Antiquity* 94:637–652.

Ringbauer H, Novembre J, Steinrücken M. 2021. Parental relatedness through time revealed by runs of homozygosity in ancient DNA. *Nature communications* 12:5425.

Rodwell S. 1984. China's earliest farmers: the evidence from Cishan. *Bulletin of the Indo-Pacific Prehistory Association* 5:55–63.

Rubinacci S, Ribeiro DM, Hofmeister RJ, Delaneau O. 2021. Efficient phasing and imputation of low-coverage sequencing data using large reference panels. *Nature Genetics* 53:120–126.

Tao L, Yuan H, Zhu K, Liu X, Guo J, Min R, He H, Cao D, Yang X, Zhou Z. 2023. Ancient genomes reveal millet farming-related demic diffusion from the Yellow River into southwest China. *Current Biology* 33:4995–5002.

Wang C-C, Yeh H-Y, Popov AN, Zhang H-Q, Matsumura H, Sirak K, Cheronet O, Kovalev A, Rohland N, Kim AM. 2021. Genomic insights into the formation of human populations in East Asia. *Nature* 591:413–419.

Wang H, Yang MA, Wangdue S, Lu H, Chen H, Li L, Dong G, Tsring T, Yuan H, He W, et al. 2023. Human genetic history on the Tibetan Plateau in the past 5100 years. *Sci. Adv.* 9:eadd5582.

Wang T, Wang W, Xie G, Li Z, Fan X, Yang Q, Wu X, Cao P, Liu Y, Yang R. 2021. Human population history at the crossroads of East and Southeast Asia since 11,000 years ago. *Cell* 184:3829–3841.

Wen J, Zheng Z, Gong M, Li D, Hu S, Cai Y, Wang Y, Nanaei HA, Zhang N, Yu T. 2022. Ancient genomes reveal the genetic inheritance and recent introgression in Chinese indigenous pigs. *Science China Life Sciences* 65:842–845.

Wu R, Cui Y, Guo R, Jin G. 2017. Results and Analysis of the 2015 Trial Excavation and Flotation at the Dashuigou Site. *Agricultural Archaeology*:13–20.

Wu Z, Chen C, Han Y, Liu Z, Li Q. 2020. Research on the faunal remains of the Xitucheng City Site, Kangbao, Hebei Provience. *Cultural Relics in Southern China*:126–133.

Wu Z, Zhang H. 2023. Research on Animal Remains Excavated from the Eastern Han Mausoleum Site M722 at Zhucang in Luoyang. *Cultural Relics in Southern China*:128-134,127.

Xu Y, Tan Y, Wu X, Xu G, Luo X, Xie X, Xie L, Xie H, Xing L, Feng C, et al. 2019. Excavation of the Shikouzi Site in Zhungeer Banner, Inner Mongolia Autonomous Region. *Wenwu Chunqiu*:34–48.

Xue Y, Dal Martello R, Qin L, Stevens CJ, Min R, Fuller DQ. 2022. Post-Neolithic broadening of agriculture in Yunnan, China: Archaeobotanical evidence from Haimenkou. *Archaeological Research in Asia* 30:100364.

Yan H, Zhang H, Lu Q, Song, Yuntao, Liu J. 2011. Imperial Tomb Complex of the Eastern Han Dynasty at the Zhucang Site, Mengjin, Luoyang. *Chinese Antiquity*:4–32.

Yang B, Cui L, Perez-Enciso M, Traspov A, Crooijmans RPMA, Zinovieva N, Schook LB, Archibald A, Gatphayak K, Knorr C, et al. 2017. Genome-wide SNP data unveils the globalization of domesticated pigs. *Genet Sel Evol* 49:71.

Yang J, Zhang D, Yang X, Wang W, Perry L, Fuller DQ, Li H, Wang J, Ren L, Xia H. 2022. Sustainable intensification of millet–pig agriculture in Neolithic North China. *Nature Sustainability* 5:780–786.

Yang MA, Fan X, Sun B, Chen C, Lang J, Ko Y-C, Tsang C, Chiu H, Wang T, Bao Q, et al. 2020. Ancient DNA indicates human population shifts and admixture in northern and southern China. *Science* 369:282–288.

You Y, Chen X, Hein A, Qin C, Zhao Y, Zhang J, Liu T, Fan W, Yuan G. 2024. Pig domestication and human subsistence at the early Neolithic site of Guanjia (6100–5500 BC), Central China. *Archaeol Anthropol Sci* 16:40.

Yu C, You Y, Luo J, Ruan Q. 2022. The Late Bronze Age pastoralist settlement at Halehaxite in the Tianshan Mountains, Xinjiang, China, a zooarchaeological perspective. *Journal of Archaeological Science: Reports* 45:103595.

Zhang M, Liu Y, Li Z, Lü P, Gardner JD, Ye M, Wang J, Yang M, Shao J, Wang W. 2022. Ancient DNA reveals the maternal genetic history of East Asian domestic pigs. *Journal of Genetics and Genomics* 49:537–546.

Zhang Q, Hou Y, Li X, Styring A, Lee-Thorp J. 2021. Stable isotopes reveal intensive pig husbandry practices in the middle Yellow River region by the Yangshao period (7000–5000 BP). *PLoS One* 16:e0257524.

Zhang W, Yang M, Wang Y, Wu X, Zhang X, Ding Y, Yin Z. 2020. Genomic analysis reveals selection signatures of the Wannan Black pig during domestication and breeding. *Asian-Australasian journal of animal sciences* 33:712.

Zhong H, Li X, Wang W, Yang L, Zhao Z. 2020. Preliminary research of the farming production pattern in the Central Plain area during the Miaodigou Period. *Quaternary Sciences* 40:472–485.

Zhou Y, Wang Z, Zhang J. 2000. The Neolithic Site at Xuecheng in Gaochun County, Jiangsu. *Chinese Archaeology*:1–20.

Zong T, Guo X, Liu H, Zhang X, Li Y. 2021. Animal remains reveal the development of subsistence economy from prehistory to Qin-Han periods in the Guanzhong region: Evidence from the Gongbeiya site in Xi'an. *Quaternary Sciences* 41:1445–1454.

Legends of supplementary tables S1, S2, S4 and S5

Supplementary table S1. Information of new ancient and published genomes used in this study. (A) New ancient samples used in this study. (B) The information of all pig samples screened for next generation sequencing. (C) Published SRA Data used in this study. (D) Published ancient mitochondrial genome nucleotide sequences.

Supplementary table S2. The results of the outgroup f_3 and f_4-statistics. (A) The results of the outgroup f_3-statistics analysis conducted by ancient archaeological sites. (B) The results of the f_4-statistics analysis conducted by ancient archaeological sites. (C) The results of outgroup f_3-statistics analysis assessing the genetic affinity between all ancient or modern groups. (D) The results of f_4-statistics analysis indicating that Early North members (Cishan/Miaodigou/SS14), compared to other populations, have significant gene flows with Late North. (E)The results of the f_4-statistics analysis indicating extra affinity between Late North and Miaodigou compared to other North-proto members. (F)The results of the f_4-statistics analysis testing the relationship between Southwest populations and ancient Chinese domestic pigs. (G) The results of f_4-statistics analysis assessing the affinities between modern domestic pigs with Late North in comparison to other ancient populations.

Supplementary table S4. The results of qpAdm modeling. (A) The results of qpAdm modeling of domestic pigs in China (excluding Southern China) with Miaodigou, Haimenkou, West commercial. (B) The results of qpAdm modeling of domestic pigs in China (excluding Southern China) with Late North, Haimenkou, West commercial. (C)

The results of qpAdm modeling of domestic pigs in Southern China. (D) The results of using Sumatran as a source to model all modern Chinese domestic pigs and the ancient domestic pig from Haimenkou.

Supplementary table S5. The analytical results on the *MC1R* gene and black coloration in Sus scrofa. (A) Genotypes of the causative mutation (Chr6:181883 A>G) in MC1R for black coloration of Chinese domestic pigs. (B) Frequency of the alternative allele in *MC1R* gene (Sscrofa11 Chr6:181883 A>G) for black coloration in Asian domestic pigs estimated using a maximum likelihood method (95% CI). (C) Allele frequencies calculated with imputed data. (D) Ancient Samples used for imputation. (E) Coordinates of the eight Mutations and Phased Haplotypes in Imputed Sequences Referenced to Sscrofa11 Chr6.
